# Fast-Forward: Automatic Assignment and Assessment of Bonded Parameters for the Martini Force Field

**DOI:** 10.1021/acs.jcim.6c01107

**Published:** 2026-08-18

**Authors:** Christopher Brasnett, Maximilian Fidlin, Thilo Duve, Sebastian Thallmair, Siewert J. Marrink, Fabian Grünewald

**Affiliations:** † Groningen Biomolecular Sciences and Biotechnology Institute, University of Groningen, 9747 AG Groningen, The Netherlands; ‡ Heidelberg Institute for Theoretical Studies (HITS), Schloss-Wolfsbrunnenweg 35, 69118 Heidelberg, Germany; § Institute of Pharmacy and Molecular Biotechnology (IPMB), 9144 Heidelberg University, Im Neuenheimer Feld 364, 69120 Heidelberg, Germany; ∥ 505684 Frankfurt Institute for Advanced Studies, Ruth-Moufang-Straße 1, 60438 Frankfurt am Main, Germany; ⊥ Faculty of Biochemistry, Chemistry and Pharmacy, Goethe University Frankfurt, Max-von-Laue-Straße 9, 60438 Frankfurt am Main, Germany

## Abstract

Coarse-grained molecular dynamics simulations of bio- and macromolecular systems offer a method of accessing otherwise unobtainable time and length scales, compared to atomistic simulation techniques. However, a limiting step is often the generation and validation of the coarse-grained models. Here, we describe a new software package, Fast-Forward, which aids parametrization of models for the widely used Martini coarse-grained force field. In comparison to other similar packages, Fast-Forward offers a system-agnostic suite of tools to parametrize molecules of any size, existing in any environment. It achieves this while maintaining ease of use and the use of interoperable file formats from the Martini software ecosystem. Through its three subprograms, the package offers tools for trajectory mapping, parameter generation, and model validation. We demonstrate the potential of the package for several different use cases, from small biological molecules (glutathione and glutathione disulfide) to models of synthetic polymers (poly­(methyl methacrylate), PMMA).

## Graphical abstract

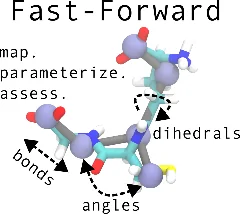

## Introduction

Molecular dynamics (MD) simulations have found widespread application in addressing molecular scale questions otherwise inaccessible to experimental techniques. However, for many applications, the size and time scales of molecular processes prohibit the use of all-atom force fields, as the degrees of freedom required for simulation are too large even for modern computing infrastructure. To overcome this challenge coarse-grained (CG) simulation methods are frequently used, where atoms are grouped together into so-called beads, whose interactions are suitably calibrated to reproduce realistic behavior.^1–4^ Among CG force fields, the Martini force field has attracted particular popularity for its ease of use and widespread applicability, covering lipids, proteins, sugars, polymers, and many more molecules besides.^5–12^

In the Martini force field, two to four nonhydrogen atoms together with their bound hydrogens are mapped to a single bead. The most recent version, Martini 3, has validated models for a wide selection of compounds that are available for the community through the webportal or the MArtini Database (MAD).^13–15^ To obtain simulation parameters for a new molecule, researchers are recommended to follow specific guidelines.^5,11,16^ First the mapping is decided based on similarity to existing molecules. Subsequently, a bead type needs to be assigned from a preparameterized set of beads. The bead type defines all the nonbonded interactions in the system, i.e., the strength of the Lennard-Jones potential as well as the charges taken into account in the Coulomb potential that are only considered for ions or ionic groups. The choice of bead types is typically validated by matching thermodynamic reference data such as free energies of transfer between water and organic solvents. Once the bead types are assigned, the bonded interactions (bonds, angles, dihedrals) need to be generated by fitting to an atomistic reference simulation. Bonded interactions within the molecule may then be tuned for fidelity and stability in subsequent optimization simulations. While in general a strict center of geometry mapping scheme should be followed, bonded interactions can be further tuned to match the solvent accessible surface area (SASA) obtained from atomistic simulation and/or experimental data available.^10,17^ Consequently, the parameterization process consists of three elementary steps: (1) mapping, (2) bead assignment, and (3) matching bonded interactions.

Previously, several packages have been developed to aid the user community in parametrizing molecules for the Martini force field. Each package promises varying levels of automation for the elementary parameterization steps and refinement processes. Overall, they fall into one of three classes. Class I consists of those packages historically developed with the aim to map and fit bonded interactions. These tools include, among others, PyCGTool and Bartender.^18,19^ They have in common that they map a reference simulation and convert it to bonded parameters by a dedicated fitting procedure and the subsequent Boltzmann inversion of the fitted parameters.^20,21^ Some packages such as Bartender also have utilities to automatically run the required simulations. Bartender is also special in the sense that it uses quantum mechanics (QM) data as reference, rather than classical all-atom simulations. QM reference data is also used by another recent tool, which uses Fast Fourier Transform methods to quantitatively match dihedral bonded distributions for both all atom (AA) and CG parameterization.^22^ Class II packages, in contrast, not only map and match bonded interactions but they also iteratively run CG simulations to refine initial parameters. In principle, they can also match other target properties, which allows to refine bonded interactions in combination with bead type assignments. For example, SwarmCG and CGCompiler both utilize particle swarm algorithms to navigate the high dimensional parameter space.^23–26^ Similarly, jax-martini uses gradient-based optimization methods to optimize models against both microscopic (e.g., bonded parameters) and macroscopic (e.g., interfacial area per lipid) targets.^27^ Class III tools follow the spirit of automated topology builders known from all-atom force fields. Starting from a molecule definition they directly generate a mapping and the corresponding bead-type assignment. Subsequently, bonded parameters are selected by a rule-based algorithm without taking any reference simulation into account. CG_param, AutoMartini, and Martini Mapper are examples of Class III tools, the first two having both recently been updated to Martini 3.^28–30^

Each class of tool has its specific advantages and disadvantages. For example, while Class II tools can result in very accurate parameters, they are typically slow and require much manual tuning. This knowledge requirement makes them especially difficult to use for nonexperts. On the other end of this spectrum are Class III tools that are fast and easy to use. However, they can produce topologies of questionable accuracy and are limited to small molecules. AutoMartini is fundamentally limited to molecules no larger than 24 heavy atoms, for example. Finally, Class I tools offer a compromise between easy to use and accuracy. At the same time, they still require manual bead assignment and cannot optimize global properties.

Despite the many successes of existing Class I tools, they all struggle when it comes to generating bonded parameters for large complex macromolecules and polymers. In particular, none of them allows either a modular (e.g., residue or fragment based) definition of the mapping or utilizes one of the standardized mapping file formats, which have been established in the Martini ecosystem of tools.^31–33^ Instead they utilize index based definitions, which are error prone and need to be adjusted when switching between reference atomistic resolution force fields. The compatibility of file formats is especially important for advancing multiscale and high-throughput approaches, which rely on integrating several different tools such as martinize2 and Backward in the same workflow. Some tools only handle a single molecule at a time, which complicates taking into account collective behavior, for instance in a polymer melt or lipid membrane. Other tools are not suitable to fit dihedral parameters and virtual sites, both important structural components of macromolecules in the Martini 3 force field.

Another shortcoming of the current procedures is the lack of a standardized evaluation protocol for assessing whether the bonded interactions have been fit sufficiently well. Assuring the quality of these fits is particularly important, since many Martini models are generated by nondevelopers and Martini 3 is more sensitive than Martini 2 with respect to molecular geometry.

Thus, there is currently a vacancy in the Martini software ecosystem for a tool aiding parameterization of molecules at a variety of scales, using modular file formats compatible with the rest of the ecosystem, and enabling user debugging during model optimization.

In this work, we present the Fast-Forward Python package to address this deficit in parameterization tools currently available to the community. The design philosophy of Fast-Forward is rooted in the simplicity of Class I tools but integrates aspects of the feedback loop used in Class II tools as well. It is not intended to be an automated optimizer in the spirit of Class II tools, nor automatically generating bonded parameters as Class III tools would. Instead, it offers a lightweight and extensible ecosystem-compatible framework to aid the high-throughput and rapid parameterization of molecules of, in principle, any size.

To show the range of functionality of the package, we present several showcase examples of how molecular parameterization can be performed with the Fast-Forward package. These examples show how relatively simple molecules can be parametrized with a view to more complex systems in the future. Beyond the examples presented in this work, earlier versions of Fast-Forward were used to aid the development of the Martini3-IDP force field and the Martini 3 Metabolome library.^34,35^ These further examples show how the package could in principle already be used for high-throughput studies or heterogeneous polymer parameterization. However, it may not be suitable for all systems and has not (for example) been tested on crowded multicomponent systems. Additionally, the final parameterization is limited to a number of potentials for each bonded interaction, which may not be appropriate in all cases.

At its core, Fast-Forward maps, fits, and assesses bonded interactions for CG molecules. To allow for standardized assessment of bonded interactions, we further define a hierarchical scoring strategy. This strategy is applicable to any Martini molecule parameterization. Fast-Forward builds on the current ecosystem of tools for the Martini force field, using standardized file formats such as. map files for residue mapping. The package is lightweight and designed to be easy to use. Herein, we describe the design of the package and its subprograms and demonstrate its use in parametrizing a range of molecules for the Martini force field. We finally discuss the advantages of Fast-Forward compared to other existing software packages.

## Fast-Forward

### Package design

Fast-Forward is designed to complement the standard parameterization workflow for molecules according to the Martini force field as described above. The workflow is divided between three subprograms of the Fast-Forward package, ff_map, ff_inter, and ff_assess. The workflow is shown in Figure 1, demonstrating the required input data at each stage, and how the outputs from one subprogram feed into subsequent ones. In keeping with the design philosophy of much of the rest of the Martini ecosystem, Fast-Forward is intentionally lightweight in design, with few hard dependencies (namely, MDAnalysis, LMfit, and Vermouth).^31,36–38^ Core parts of the package such as the interaction fitting are also designed to be exposable for custom analyses as may be desired by users.

**Figure 1:**
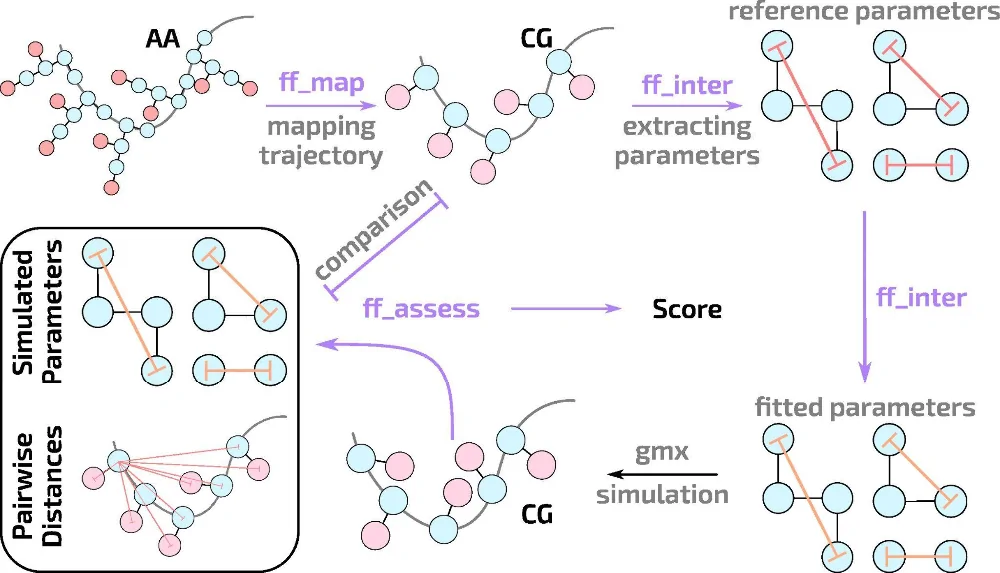
**Workflow of the Fast-Forward package.** Using an atomistic reference trajectory, a set of CG parameters is fitted from a mapped pseudo-CG trajectory and their performance quantified after Gromacs (gmx) simulation. Quantification of the fitted parameters is done on a local and global scale, yielding an interaction (local) and distance score (global). A technical presentation of the package, along with the format of input data types, is shown in Figure S1.

### Subprogram ff_map

The first subprogram, ff_map, is the mapping program of fast-forward. The subprogram first reads a (waterless) atomistic trajectory and converts it to a pseudo-CG one using center of geometry mapping information obtained from a second input. Mapping information is derived from a Backward-style mapping file, a format associated with the Vermouth-Martinize package. Alternatively, a CGSmiles string, specifying a multiresolution molecular description, may be provided.^33^ We provide examples of both of these formats and their use in the SI, Section 5. The mapping format is designed with flexibility in mind, for example, by permitting weighted mapping within a bead to indicate relative importance of atoms to the final position. This extends to the reconstruction of implicit hydrogen atoms from united-atom force fields using the buildH package.^39^ The mapping format even allows for cross-residue mappings, functionality that may find use during the parameterization of complex heterogeneous macromolecules. Backward-style mapping formats are used by the martinize2 tool to map protein structures to CG resolution. The consistent use of this format across the Martini software ecosystem could therefore better support the *in situ* coarse-graining of ligands with proteins.

As discussed above, one feature missing from many parameterization packages is the functionality to account for collective behavior. In comparison, ff_map is not limited to a single molecule or residue type, and the file format, in fact, allows for entire systems consisting of multiple molecules or residues to be converted with a single command. The resulting pseudo-CG trajectory shows the targeted behavior of the CG trajectory obtained with the CG model that is to be designed. An example of this is shown for the molecule glutathione (GSH) in Figure 2A,B, where the atomistic structure has had a possible CG equivalent superposed.

**Figure 2:**
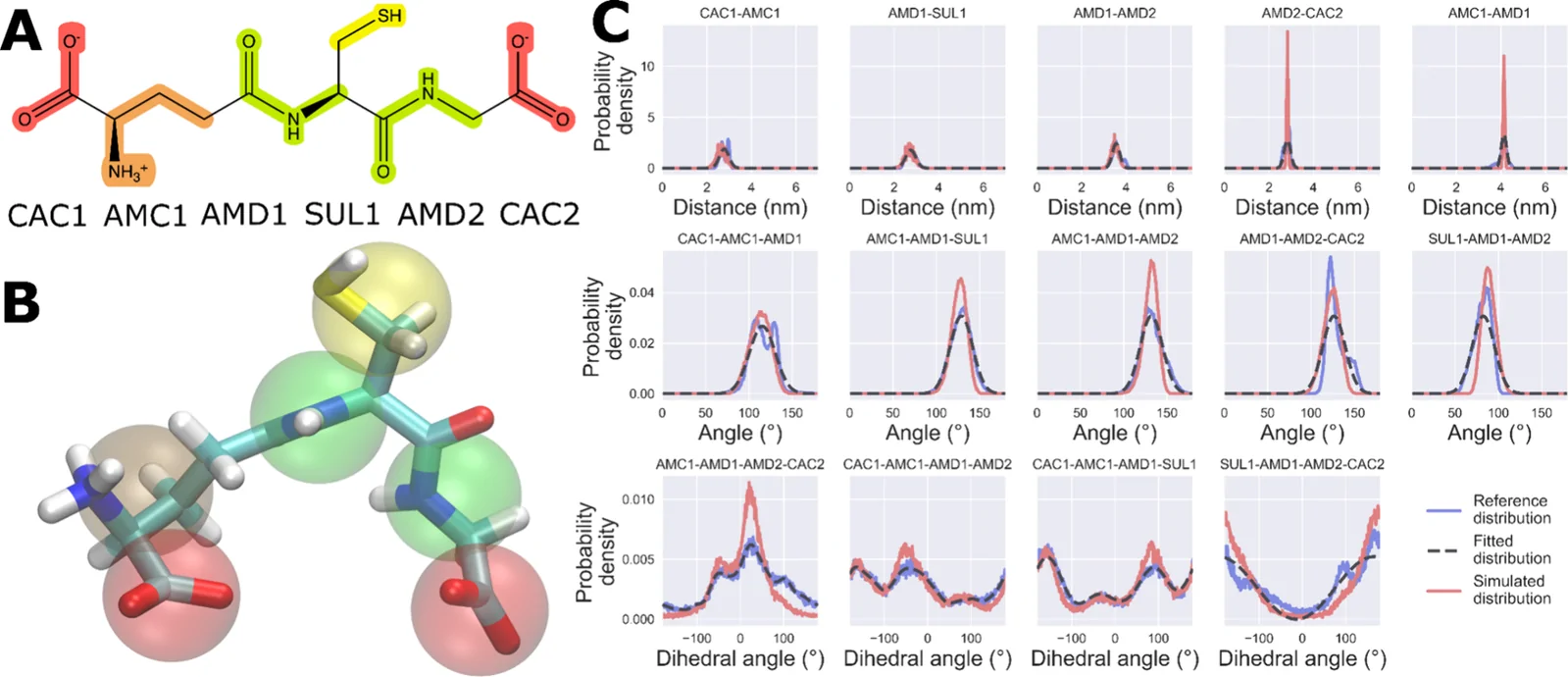
**Parameterization of GSH using the Fast-Forward workflow.** A,B) the mapping of the atomistic structure to CG resolution. A) atoms are grouped into CG beads and allocated a name. B) The atomistic trajectory is mapped to a pseudo-CG one using ff_map. C) Using ff_inter, the bonded interactions of the pseudo-CG trajectory are analyzed and distributions generated (blue) and fitted (dashed black). A CG simulation is then run using the new parameters, and the resulting distributions (red) compared to the reference pseudo-CG distributions.

### Subprogram ff_inter

The second subprogram described in Figure 1, ff_inter, uses pseudo-CG trajectories to determine the distributions of the bonded interactions within molecules. By providing reference molecular topologies with annotated interactions, the subprogram rapidly calculates bonded interactions and pairwise distances, taking advantage of computational acceleration via the numba package.^40^ Distributions of interactions are then calculated from time-series data.

The principal feature of the subprogram is the generation of molecular topologies based on the fitted interaction data. While such a feature is also available in many of the tools described above, the ff_inter subprogram differs by providing more comprehensive parameter estimation. The ff_inter subprogram fits not only bonds and angles but also dihedrals of arbitrary multiplicities and virtual sites. The annotation syntax of input topologies also allows for identical groups of beads to have their interactions fitted together, to provide a systematic description of the bonded interaction behavior.

A further advantageous feature that ff_inter has over similar programs is the ability to generate a full description of interactions from a minimal molecular graph. That is, by providing simply the bonds in an input topology file, ff_inter can generate all possible angles and dihedrals required for a full description. Importantly, ff_inter will check the constraints in the molecular topology and issue a warning if the constraint coupling could potentially lead to artificial temperature gradients in the simulation system.^41^ The LINCS order necessary for resolving the constraints can be estimated as well, in order to help user optimization of mapping schemes, or identify potential numerical stability issues of the model during simulation.^41–43^

As with ff_map, the ff_inter subprogram also allows for interactions of whole systems to be analyzed based simply on the input topologies given. For example, the interactions of a single dopant lipid in a host membrane could easily be isolated for parameterization. One difference that ff_inter has against Class II packages is that it does not attempt to iteratively improve the initially generated parameters; subsequent refinement is left to users.

An example of the fitting procedure is shown in Figure 2C for the pseudo-CG trajectory of the GSH molecule mapped above. While - at least in this case - the bond lengths and angular distributions can be described as simple Gaussian distributions, the dihedrals cannot. For three of the dihedrals identified along the molecule, a convolution of several periodic functions was used to fit the obtained distributions. In the final case (SUL1-AMD1-AMD2-CAC2), a single periodic function has been determined to be the best description of the data, as a more complex convolution of functions would likely result in numerically unstable parameters. Note that here, the subprogram ff_inter allows for the automatic identification of the five angles and four dihedrals from a minimal GROMACS topology file consisting of the bonds only.

### Subprogram ff_assess and hierarchical scoring

While the Martini community has developed a great many models for simulation, the fidelity of the models to their reference data has rarely been either objectively measured or reported. Often, a comparison of a CG development model to its pseudo-CG reference has been informally assessed as a sufficiently close match, before moving on with the next stage of parameterization. The third subprogram, ff_assess, is designed to provide a more objective measurement of the similarity between CG and pseudo-CG trajectory. The subprogram takes 1) a simulated CG trajectory, 2) the associated topology of the molecule simulated, and 3) the distributions calculated previously by ff_inter to calculate a summary score comparing distributions. The interaction score uses the Hellinger distance *H*(*P*, *Q*), defined in eq 1:

$$H\left(P,Q\right)=\frac{1}{\sqrt{2}}\sqrt{\overset{k}{\underset{i=1}{\sum }}{\left(\sqrt{p_{i}}-\sqrt{q_{i}}\right)}^{2}}$$ 1

Where *P* and *Q* are two discrete probability distributions, each of size *k*. The Hellinger distance is a score between 0 (completely identical) and 1 (no overlap at all) determining the similarity of two distributions. It was chosen as the metric for assessment for its simplicity of calculation and value range of [0, 1], allowing for simple and rapid assessment of a model’s quality. It is chosen, as opposed to the Wasserstein metric used in Swarm-CG and CGCompiler, because the Wasserstein distance is very sensitive to outliers - unmatched tails of distributions with a small probability mass can lead to a large increase in the Wasserstein distance. This effect is less drastic for the Hellinger distance. The Hellinger distance is calculated for each bonded term in the molecular topology, and averaged to result in a single score to indicate the overall likeness between the pseudo-CG trajectory and the newly simulated one.

While this interaction score allows for the assessment of the bonded interactions defined in the molecular topology, it does not ensure that the bonded interactions chosen are suitable or sufficient to accurately represent the molecule’s conformational space. In order to get a more accurate and quantitative view of the overall behavior of the molecule, ff_assess additionally reports the distance score *S*(*P*, *Q*). It compares all internal pairwise bead distances between the CG and pseudo-CG trajectories and reports a score based on the Hellinger distance but also includes the difference in mean between the two distributions, as defined in eq 2:

$$S\left(P,Q\right)=0.7H\left(P,Q\right)+0.3\min\left(1,\frac{\left|{\mathrm{x̅}}_{P}-{\mathrm{x̅}}_{Q}\right|}{2s_{P}}\right)$$ 2

where x̲ is the respective sample mean and *s*_*P*_ is the standard deviation of the reference distribution. The additional term is introduced to ensure agreement in the mean alongside overall distributional similarity (Figure S2). Weightings for the Hellinger distance between 0.5 and 0.9 were tested and a weighting of 0.7 was chosen to prioritize distributional similarity while still accounting for differences in the mean. This weighting is user-configurable and may be refined as further validation data becomes available. As before, the score ranges from 0 (identical) to 1 (no overlap).

The pairwise distance score reported is the mean of the score *S*(*P*, *Q*) for all pairwise bead distances. Additionally, a matrix of the scores is provided, which can be used to track down potential issues in the molecular topology. For example, missing interactions can manifest as deviations in pairwise distances, which are calculated with a distance score. Thus, a high distance score may indicate that the chosen bonded terms require refinement or that additional terms should be introduced to better represent the system of interest. For example, if the distances between beads 1 and 4 are off, then the dihedral 1–2–3–4 might need to be included. By giving users a single score, the subprogram ff_assess aims to result in a standard reporting method for the parameterization of new molecules for the Martini force field to indicate the validity to the wider community.

To establish a reference range for this metric, we evaluated six molecules from the Bartender flexible molecules data set (M21–M25 and Pitolisant (PITO)),^19^ along with GSH, glutathione disulfide (GSSG), and poly­(methyl methacrylate) (PMMA) presented in this work. The respective mappings, distance score matrices, and bonded distributions are shown in Figures S2–S10. These systems were parametrized using the Fast-Forward workflow. The molecules from the Bartender data set were parametrized using predefined topologies, i.e. the set of bonded interactions for the molecule were not generated from the minimal molecular graph as described above. The resulting scores are shown in Figure 3 and range between 0.15 and 0.38, suggesting a practical target range for this measure. Based on these results, scores below 0.3 can be considered good, while values between 0.3 and 0.5 indicate acceptable but improvable parametrizations. These thresholds should be regarded as orientation guides rather than strict cutoffs.

**Figure 3:**
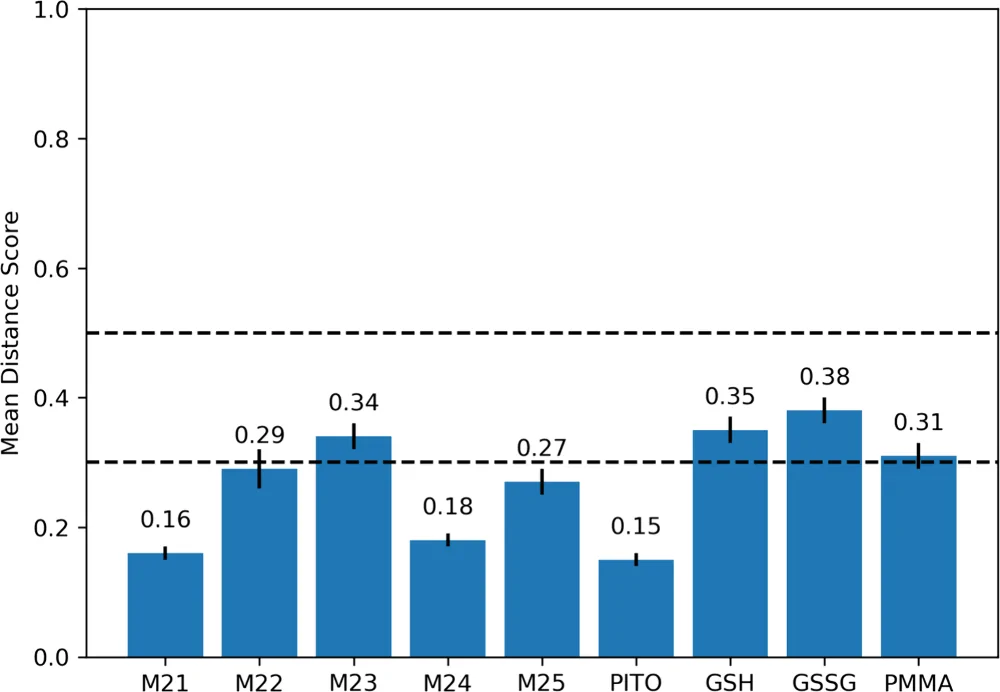
Mean distance scores of example molecules parametrized in this work. Cutoffs for good (<0.3) and acceptable (<0.5) scores are indicated. Error bars indicate the standard error of the mean.

## Showcases

### Parameterization of Small Molecules

GSH is a significant antioxidant gamma-linked tripeptide found in cells, responsible for cell redox states.^44^ GSH has not previously been parametrized in any version of the Martini force field. Based on a 1 μs all-atom simulation of GSH using the CHARMM36 force field, we developed a high-fidelity model of GSH using Fast-Forward. The mapping and interactions for this model are depicted in Figure 2. The topology of GSH generated by ff_inter includes four dihedrals, with two of them described by a total of 9 stacked dihedral functions of increasing multiplicity. Somewhat remarkably, this molecule was stable at CG resolution for 10 μs using a time step of 20 fs, indicating that - at least in this circumstance - complex stacked dihedrals can be stable at CG resolution with longer time steps. The final distributions of the bonded parameters are shown in Figure 2C, superposed on the pseudo-CG and fitted distributions. The mean interaction score is 0.26, indicating that the initially generated topology provides at least a useful starting point for further optimization. This score is likely further biased by the two constraints (bonds AMC1-AMD1 and AMD2-CAC2), which if implemented asbonds, would have force constants of values in excess of 10000 kJ/mol/nm^2^. The use of constraints also has a necessary perturbing effect on the distance score matrix, as noted in Figure S9. The angle parameters of the molecules could benefit from further optimization of force constants or equilibrium positions. The dihedrals all show an impressive resemblance to the mapped distributions of the atomistic simulation, even if one of them (AMC1-AMD1-AMD2-CAC2) could require a longer simulation time to converge to the fitted distribution shown previously.

As an extension of the parameterization of GSH we have also parametrized its oxidized form, GSSG. GSSG also demonstrated a perhaps surprising level of stability across a 10 μs simulation considering the multiple dihedral potentials covering the disulfide bond. Details of the mapping of GSSG, along with pseudo-CG distributions and resultant CG distributions are shown in Figures S10 and S11. These examples demonstrate that the Fast-Forward package can be used to rapidly generate high-fidelity and stable molecular topologies for the Martini force field. Note that we did not attempt to further optimize the topologies of either GSH or GSSG beyond the ones initially generated by the ff_inter subprogram, which may have lowered the mean distance score calculated by the assessment subprogram ff_assess.

### Parametrization of Polymers and Macromolecules

Polymers play a central role in applications and fundamental studies within material and medical sciences. Their emergent properties arise from complex interplays between bonded and nonbonded interactions, making accurate CG representations thereof challenging to find and often requiring extensive parameterization efforts.^45^ Here, we used Fast-Forward to generate initial guesses for bonded parameters and assessed their quality using poly­(methyl methacrylate) (PMMA) in chloroform as a minimal test system. In addition to the usual matching of bonded parameters to their reference distributions, long-range structural properties — namely, the radius of gyration (*R*_g_) and end-to-end distance (E2E) — were used as parameterization targets, as these are typically employed to validate Martini polymer models. Programs to calculate polymer property metrics from simulation are already widely available (e.g., within MDAnalysis) and so are not explicitly packaged as part of Fast-Forward. These metrics are an example of how additional system-specific knowledge can be necessary to fully validate molecules beyond simple interaction matching. Because parameter optimization depends heavily on the metric used to quantify the difference between two distributions, the influence of bonded interactions on the long-range properties was quantified through the Jensen Shannon divergence (JSD), a measure that has been used previously in this context.^46–48^

To illustrate the interplay between local bonded parameters and long-range properties, we examined the effect of using different dihedral constructions along the backbone (BB) and side chain (SC) of PMMA, as indicated in Figure 4A.

**Figure 4:**
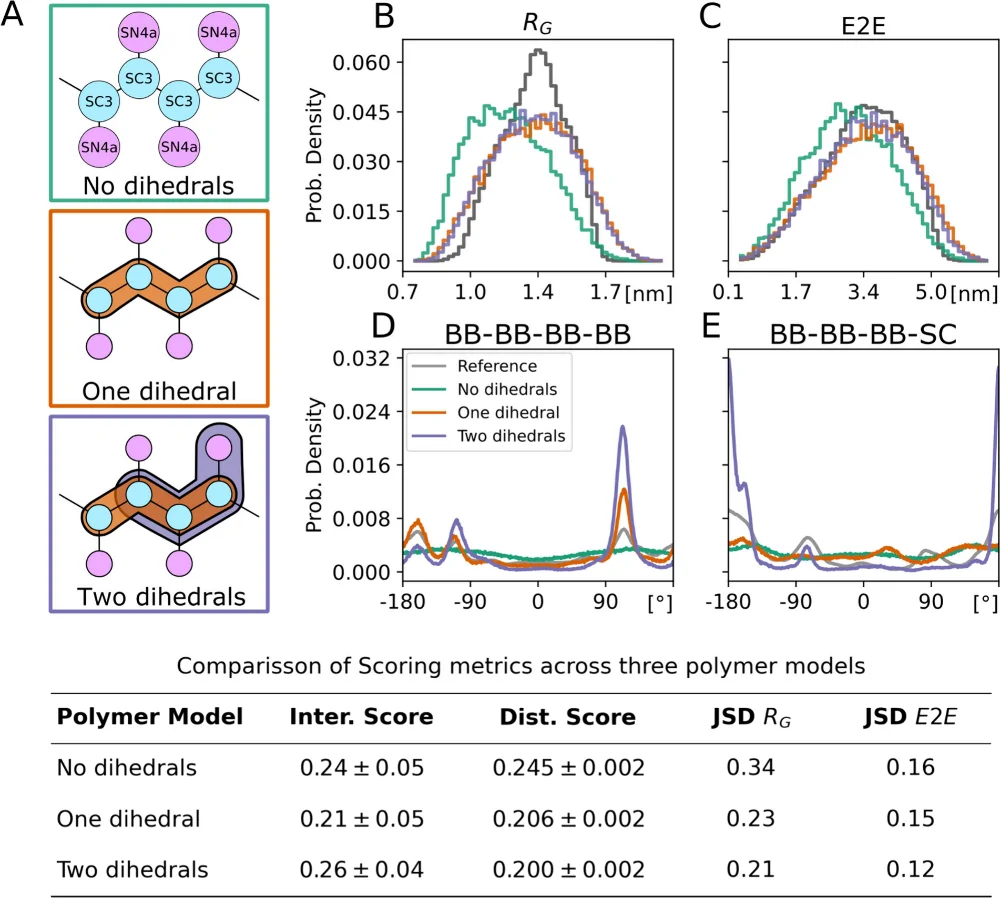
Dihedrals enable fine adjustments to parametrize macroscopic PMMA behavior. (A) Graphic description of the mapping used for all simulations, as well as three CG models: without, with one, and with two backbone dihedrals. The used dihedrals are indicated through overlapping shadows. (B) Radius of gyration distributions, the atomistic reference is shown in dark gray. The other models are displayed in different colors, corresponding to the frame color used in panel A. (C) End-to-end distance distributions. (D, E) Selected dihedral angles set progressively (BB-BB-BB-BB, BB-BB-BB-SC) for each of the polymer models.

Without any dihedrals, the interaction and distance scores generated by ff_assess suggest that the bonded parameters represent a reasonable model of PMMA (Figure S12, table in Figure 4, detailed scores for all bonded interactions in Table S1). However, it is clear from both the *R*_g_ and E2E distributions shown in Figure 4B,C that the model does not reproduce the long-range properties as faithfully. This result demonstrates that interaction and distance scores should be interpreted within the context of the parameterization target rather than treated as absolute measures of quality. Consulting additional metrics is sometimes necessary to fully verify the model performance.

To improve the agreement with these two metrics, two additional models were tested: one incorporating a backbone dihedral term (BB-BB-BB-BB) and a second that additionally included a side chain interaction term (BB-BB-BB-SC) (Figure 4A, orange and purple, respectively). The introduction of these dihedral potentials improves all three distance-based metrics (Dist. Score, *R*_g_, E2E), which means that the overall conformational ensemble is better-reproduced. The other bonded interactions are unperturbed (Figure S12). This observation is in agreement with previous studies highlighting the importance of dihedral angles in modeling polymers with Martini.

Interestingly, the model with two additional dihedral potentials has a worse interaction score for the additional dihedral angle compared to the model that does not include that dihedral angle (Supporting Information, Table S1). At the same time, only the E2E shows a larger improvement than in the previous step when going from one to two dihedrals. This analysis shows a common trade-off between improving long-range properties and perfectly matching the local bonded parameters. The origin of the problem likely stems from the choice of mapping the side chain as a single small bead, which restricts 1–3 and 1–4 distances relative to the atomistic model (Figure S13). The additional dihedral term covering the side chain compensates for this restriction, leading to an improved E2E at the expense of accurately reproducing the local dihedral angle profile. Overall, this example shows that while additional bonded parameters can improve conformational ensembles–such as in the Martini 3-IDP force field extension–there is a limit to what can be achieved.^34^ However, using the score metrics provided by Fast-Forward users can quantitatively assess these effects and make an informed decision. For PMMA, we would recommend using the model with a single dihedral and slightly worse E2E than the one with the compensation effect.

## Discussion and Conclusion

Here we have presented a new package, Fast-Forward, to aid the parameterization of new molecules. Fast-Forward achieves this through several subprograms, enabling the conversion of simulation trajectories from atomistic resolution to a CG one, and generating and assessing reference distributions. Where Fast-Forward differs from other existing packages in this regard is that it does not set out to provide a definitive or fully automated workflow to achieve this goal.

Instead, Fast-Forward is a lightweight package, rapidly deployed and easy to use, to provide high-fidelity initial parameters for users to further optimize themselves if needed. The ff_assess subprogram in particular offers a powerful tool to aid manual optimization beyond initially assigned parameters. This compares favorably with, for example, the bartender tool, which can require specialist knowledge and complex dependency management to interface with common GROMACS file formats. We also note that while the scores provide a useful heuristic for model assessment, they should be treated with caution and not used as a definitive validation of a model. The thresholds indicated in Figure 3 should be considered diagnostic guides and metrics, to be used in conjunction with system-specific knowledge and awareness that can further influence model validity. A completely faithful score will still not guarantee, for example, thermodynamic validity or numerical stability, both of which should be regarded as key targets for the Martini force field.

A second advantage of Fast-Forward over other tools is that it is not limited to the goal of parameterization. Because the mapping functionality is separated out from parameterization, users are able to investigate large, multicomponent, and heterogeneous systems, which may elucidate unique behavior that can be accounted for in the future development of CG models. While we have focused here on parameterization for the Martini force field, because the modular programs are generic, the package may be of wider interest for researchers developing other CG force fields. As the framework is sufficiently general, it would support the development of (for example) one-bead-per-amino-acid protein force fields. In principle, with good enough atomistic simulation sampling, the framework developed here could support the development of sequence-specific bonded parameters via the mapping and interaction programs for such an endeavor. The scoring metrics used in the ff_assess subprogram may have to be adapted, but the code extensibility makes this possible.

By not taking responsibility for full model optimization, the package also allows for the possibility of users imposing model-specific knowledge into topologies post hoc. The imposition of model-specific knowledge (for example, additional exclusion terms) is also significant with regard to the importance of robust testing of CG models prior to release. This ensures that–among other details such as SASA and partitioning behavior–a model is stable and does not easily experience numerical instabilities. For example, during parameterization of the Martini 3 carbohydrates, an artificial increase in the bond length was found to be necessary in order to properly capture the SASA.^10^ User-enforced contextual knowledge such as this is not captured by packages designed to simply match bond length distributions. Whereas parameterization of molecules for the Martini 3 force field should still follow the established protocols, with its integration into the rest of the modern Martini software ecosystem, there is no reason why a future fully automated pipeline for molecular parameterization could not be designed around the current functionalities of Fast-Forward.

## Methods

### Validation of Bartender Molecules

Atomistic parameters for M21–M25 and Pitolisant (PITO) from the Bartender flexible molecules data set were obtained using CHARMM-GUI’s Ligand Reader and Modeler based on the CHARMM General Force Field (CGenFF) using SMILES strings.^19,49,50^ Simulations were performed using GROMACS 2024.5.^51^ Simulation systems and topologies were generated with the CHARMM-GUI Solution Builder. Molecules were placed in a cubic box of 5.0 × 5.0 × 5.0 nm^3^ and solvated using the TIP3P water model.^50,52,53^ NaCl was added at a concentration of 150 mM, with additional ions for neutralization. The minimization and equilibration of the system followed the standard CHARMM-GUI solvation builder procedure. The system was energy minimized using steepest descent, followed by an equilibration run for 125 ps. The equilibration was followed by a production of 2 μs. Both equilibration and production were conducted in the NPT ensemble at 303.15 K with a time step of 2 fs. The pressure of the system was controlled using the Berendsen barostat for equilibration simulations and the Parrinello–Rahman barostat for production runs.^54,55^ All atomistic simulations used an isotropic pressure coupling with a reference pressure of 1.0 bar; the compressibility was set to 4.5 × 10^–5^ bar^–1^. A Nose–Hoover thermostat was used to maintain the temperature.^56^ Electrostatic interactions were calculated with the smooth particle mesh Ewald (PME) method.^57^ The molecular trajectory was then extracted using the gmx trjconv program. The system was then mapped to a pseudo-CG trajectory using ff_map and the mappings described in the Bartender publication.^19^ CG topologies were then generated using ff_inter and identical systems to the atomistic reference were generated using COBY.^58^ The systems were energy minimized using steepest descent, and equilibrated for 500 ps using a time step of 10 fs. This was followed by a 500 ns production simulation with a 20 fs time step. Electrostatics were treated with the reaction-field scheme, using a cutoff of 1.1 nm. van der Waals interactions were treated using potential-shift-verlet, using a cutoff of 1.1 nm.^59^ Pressure was 1 bar with a compressibility of 3.0 × 10^–4^ bar^–1^. Pressure was treated using the Berendsen barostat during equilibration and the Parrinello–Rahman barostat in production. The v-rescale thermostat was used to maintain temperature at 303.15 K during all simulations.

### Parameterization of Small Molecules

Parameters of GSH and GSSG in the CHARMM36 force field were obtained from the SwissParam server using SMILES strings.^60–62^ Simulations were performed using GROMACS 2024.1.^51^ The molecules were solvated at an ionic concentration of 0.15 M NaCl, with additional ions for neutralization. The system was energy minimized using steepest descent, followed by equilibration simulations for 1 ns in NVT and NPT respectively. These were followed by a 1 μs production simulation. For all simulations, the time step was 2 fs. Where pressure coupling was used, the pressure was set to 1.0 bar with a compressibility of 4.5 × 10^–5^ bar^–1^. Electrostatic interactions were treated with PME with a cut off of 1.2 nm. During equilibration the C-rescale barostat was used, and production simulations were performed using the Parrinello–Rahman barostat with isotropic pressure coupling.^55,63^ Simulation temperature was maintained at 300 K with the v-rescale thermostat.^64^

The molecular trajectory was then extracted using the gmx trjconv program. A mapping scheme was developed following the standard Martini 3 mapping rules and a file was generated using the cgbuilder server.^65^ The system was mapped to a pseudo-CG trajectory using ff_map, and initial parameters were generated using ff_inter. The mapped molecule was then simulated at CG resolution using the standard Martini 3 settings.^59^ The molecule was solvated at identical concentration to atomistic simulations (0.15 M NaCl with neutralizing counterions), energy minimized using steepest descent, and equilibrated for 10 ns. This was followed by a 10 μs production simulation. For equilibration, a 10 fs time step was used, and for production the time step was 20 fs. Electrostatics were treated with the reaction-field scheme, using a cutoff of 1.1 nm. van der Waals interactions were treated using potential-shift-verlet, using a cutoff of 1.1 nm^59^. Pressure was 1 bar with a compressibility of 3.0 × 10^–4^ bar^–1^. Pressure was treated using the C-rescale barostat during equilibration and the Parrinello–Rahman barostat in production. The v-rescale thermostat was used to maintain temperature at 300 K during all simulations.

### Parametrization of Macromolecules

The reference distributions were obtained from simulating PMMA in chloroform (trichloromethane, TCM) for 3 μs in 3 replicas and then calculating averaged properties over all of them (Figures S14 and S15). Convergence of the atomistic simulations was assessed by calculating autocorrelation functions of the *R*_g_. The polymer systems were set up using Polyply^12^ with 25 repeat units and terminal methyl groups. The parameters for PMMA and TCM were generated with Antechamber and the GAFF.^66,67^ Prior to simulation in the NPT ensemble (2 fs time step) with an isotropic Parrinello–Rahman barostat^68^ and thermocoupling via the v-rescale thermostat,^69^ the systems were NVT equilibrated for 0.5 ns and NPT equilibrated for 10 ns. NVT equilibration was performed with a 1 fs time step, using the LINCS algorithm^43^ for H-Bond constraints and the v-rescale thermostat for thermocoupling. Before switching to the Parrinello–Rahman barostat in the production simulations, the C-rescale barostat^63^ was used for pressure coupling during NPT equilibration, in addition to the settings discussed for the NVT equilibration. After simulation the trajectories were mapped to Martini resolution using ff_map and initial parameters were generated from ff_inter. The reference bonded distributions were calculated from the mapped trajectory using ff_inter. The *R*_g_ and E2E distributions were calculated using the GROMACS tool gmx polystat.^70^

At Martini resolution, the systems were prepared likewise to the atomistic protocol and simulated for 1.6 μs. The distributions of bonded parameters were calculated using ff_inter and evaluated using ff_assess. The distance and interaction scores are reported using the standard error of the mean. *R*_g_ and E2E distributions were calculated using the GROMACS tool gmx polystat.^70^

## Supplementary Material



## Data Availability

The code is available on GitHub (https://github.com/Martini-Force-Field-Initiative/fast_forward) and is installable with pip.

## Notes

Additional details on the technical workflow and organization of the Fast-Forward package, supporting figures for the validation of the scoring matrix, validation figures for molecules parametrized as part of Figure 3 in the main text, bonded distributions used in GSSG parameterization, additional validation of PMMA parameterization, and description of mapping formats (PDF)
