# Supplementary material for: Fast-Forward: Automatic Assignment and Assessment of Bonded Parameters for the Martini Force Field
Source: J Chem Inf Model. 2026 Aug 18;66(17):10982–91. doi: 10.1021/acs.jcim.6c01107 (PMC13580554; doi:10.1021/acs.jcim.6c01107)
Supplement: Supplementary file 1 [file ci6c01107_si_001.pdf]

Supporting Information for Fast-Forward: Automatic Assignment and Assessment of Bonded Parameters for the Martini Force Field

Christopher Brasnett[1], Maximilian Fidlin[2,3], Thilo Duve[4,5], Sebastian Thallmair[4], Siewert J. Marrink[1], Fabian Grünewald[2,*]

1 Groningen Biomolecular Sciences and Biotechnology Institute, University of Groningen, 9747 AG Groningen, The Netherlands

2 Heidelberg Institute for Theoretical Studies (HITS), Schloss-Wolfsbrunnenweg 35, 69118 Heidelberg, Germany

3 Institute of Pharmacy and Molecular Biotechnology (IPMB), Im Neuenheimer Feld 364, 69120 Heidelberg, Germany

4 Frankfurt Institute for Advanced Studies, Ruth-Moufang-Straße 1, 60438 Frankfurt am Main, Germany

5 Faculty of Biochemistry, Chemistry and Pharmacy, Goethe University Frankfurt, Max-von-Laue-Straße 9, 60438 Frankfurt am Main, Germany

* corresponding author, fabian.gruenewald@h-its.org

Organisation

Section 1. Page S3: technical workflow and organization of the Fast-Forward package

Section 2. Pages S4-S12: ff_assess scoring. Supporting figures for the validation of the scoring metric, and validation figures for molecules parameterized as part of Figure 3 in the main text.

Section 3. Page S13: ff_assess distributions for GSSG parameterization.

Section 4. Pages S14-S18: Validation of PMMA parameterization.

Section 5. Pages S19-S22: Documentation of mapping formats

1. Workflow

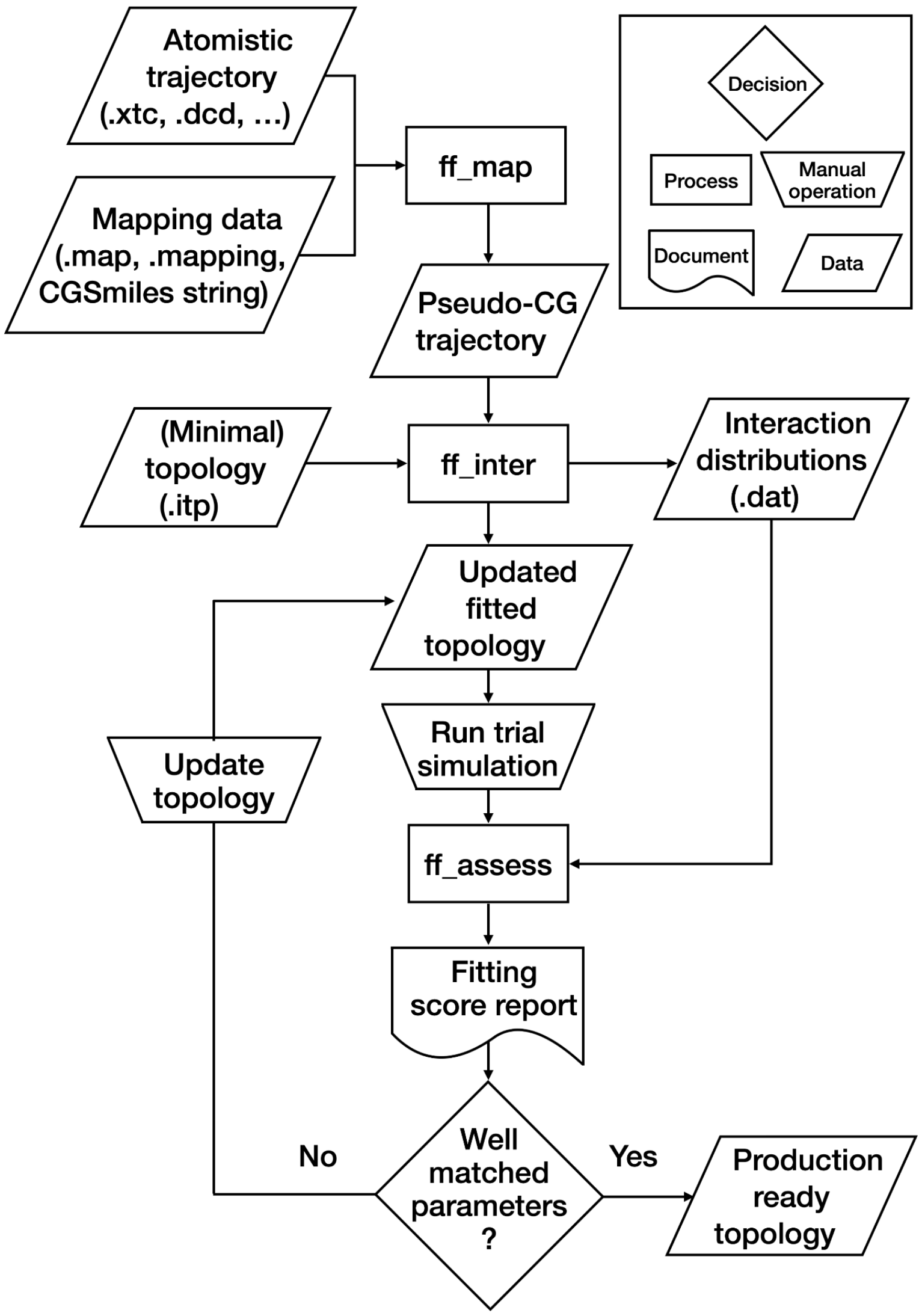

Figure S1: Workflow of Fast-Forward programs. From a set of input data (atomistic trajectories, mapping information, and preparatory coarse-grained topology files), a production-ready topology is prepared following the use of *ff_map, ff_inter,* and *ff_assess* as shown. Data and process types are described by the key in the top right of the figure.

2. Distance Score

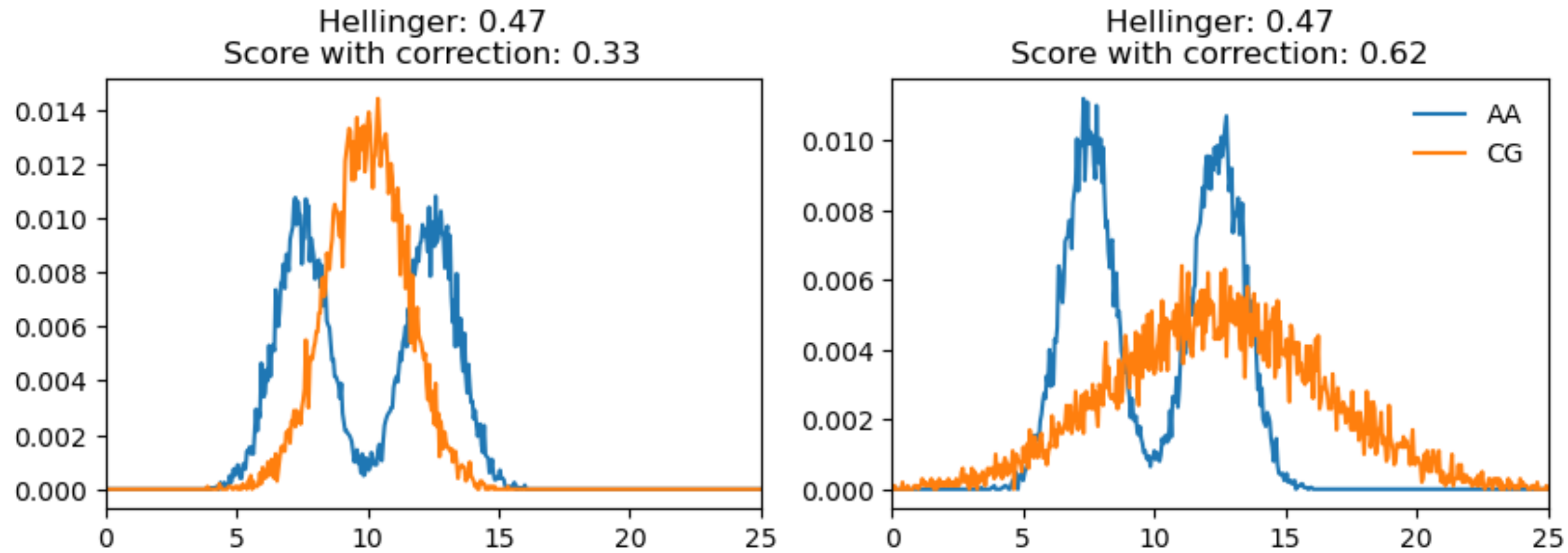

Figure S2: Distance Score correctional term. Artificial data simulation atomistic (AA) and coarse-grained (CG) distributions are shown. The correction term is introduced to emphasize agreement in the mean in addition to overall distributional similarity

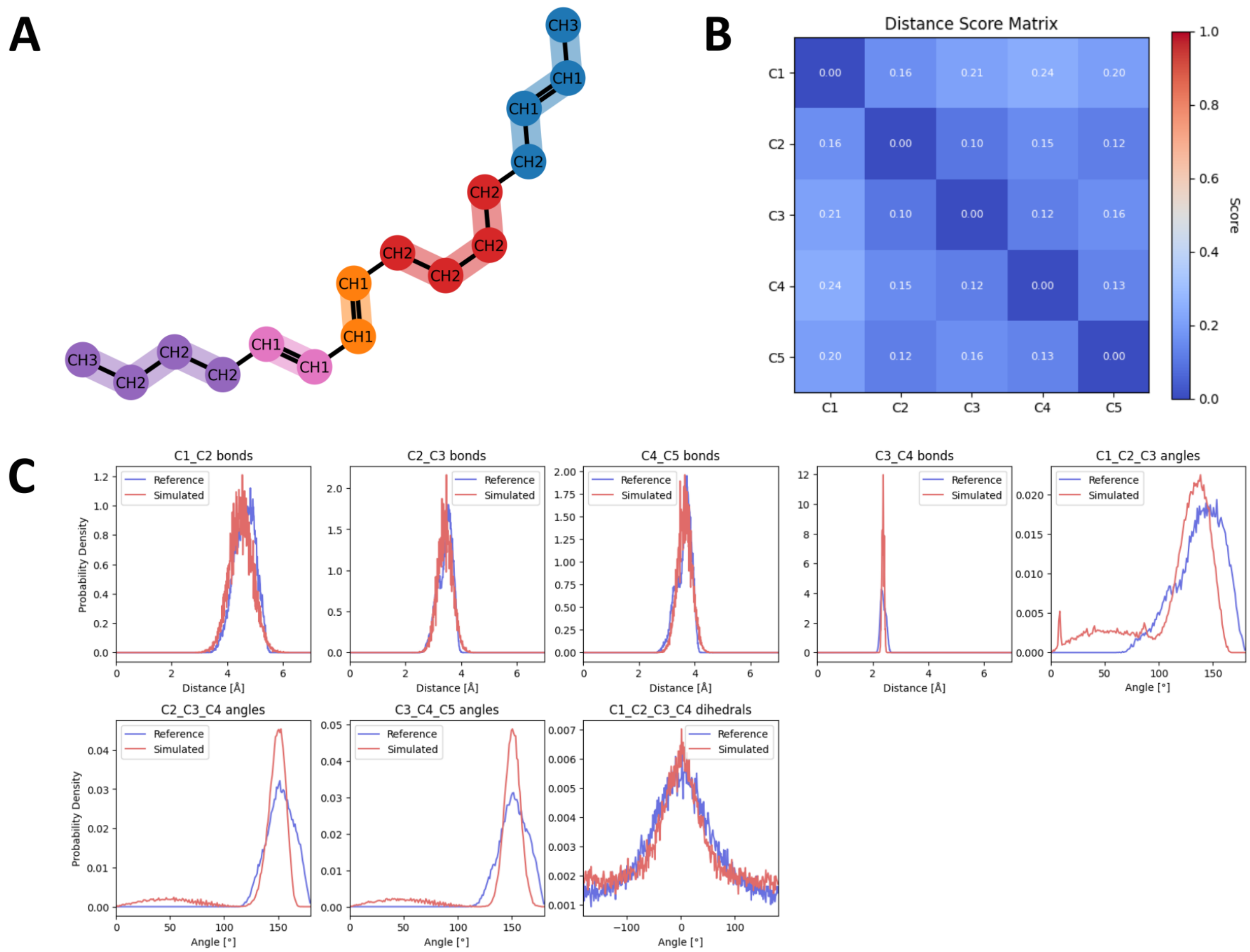

Figure S3: Parametrization of M21. **A:** the mapping of the atomistic structure to CG resolution. **B:** Distance Score Matrix showing distance score for each pairwise distance. **C:** bonded interactions of the pseudo-CG reference trajectory (blue) and distributions generated with CG Simulations (red).

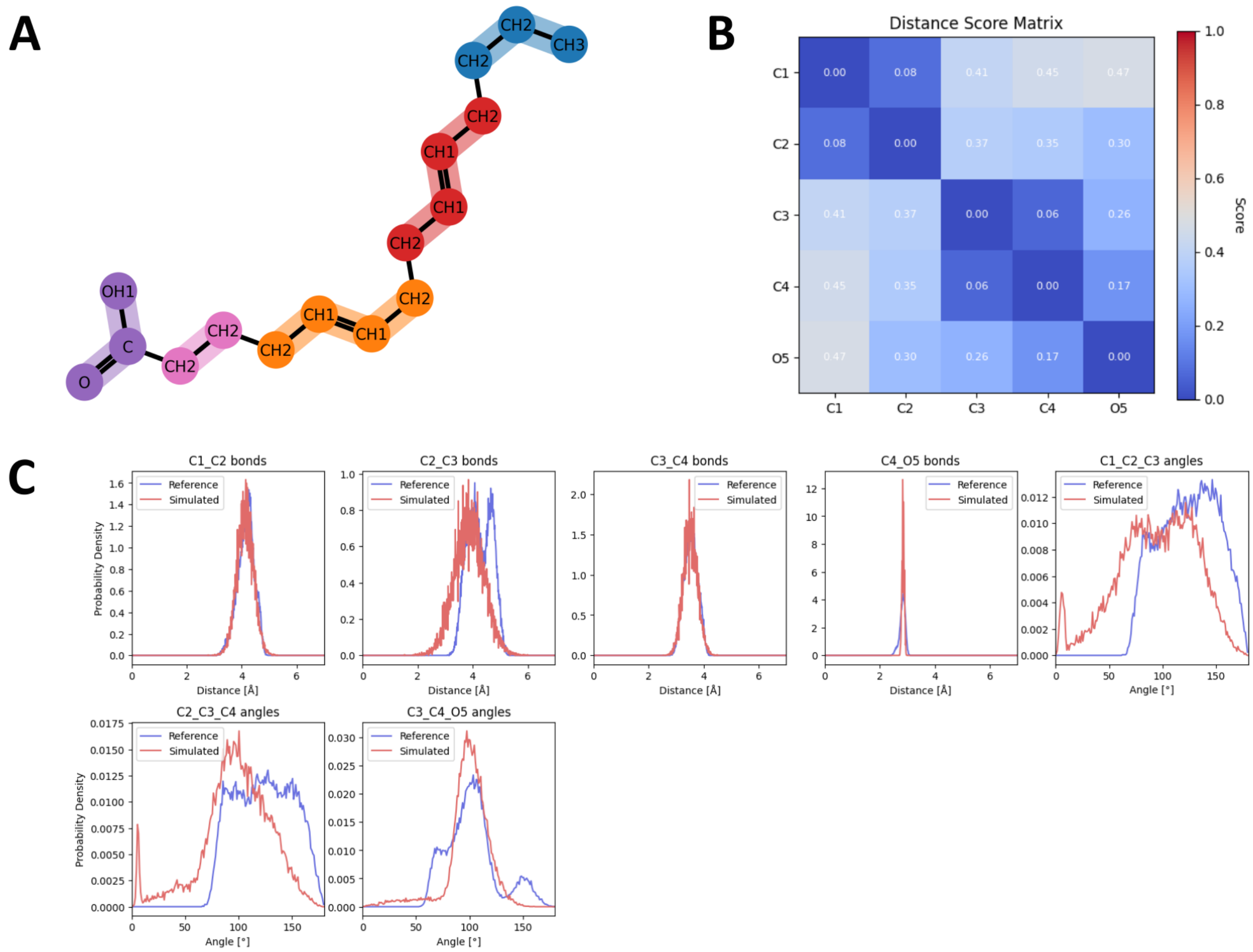

Figure S4: Parametrization of M22. **A:** the mapping of the atomistic structure to CG resolution. **B:** Distance Score Matrix showing distance score for each pairwise distance. **C:** bonded interactions of the pseudo-CG reference trajectory (blue) and distributions generated with CG Simulations (red).

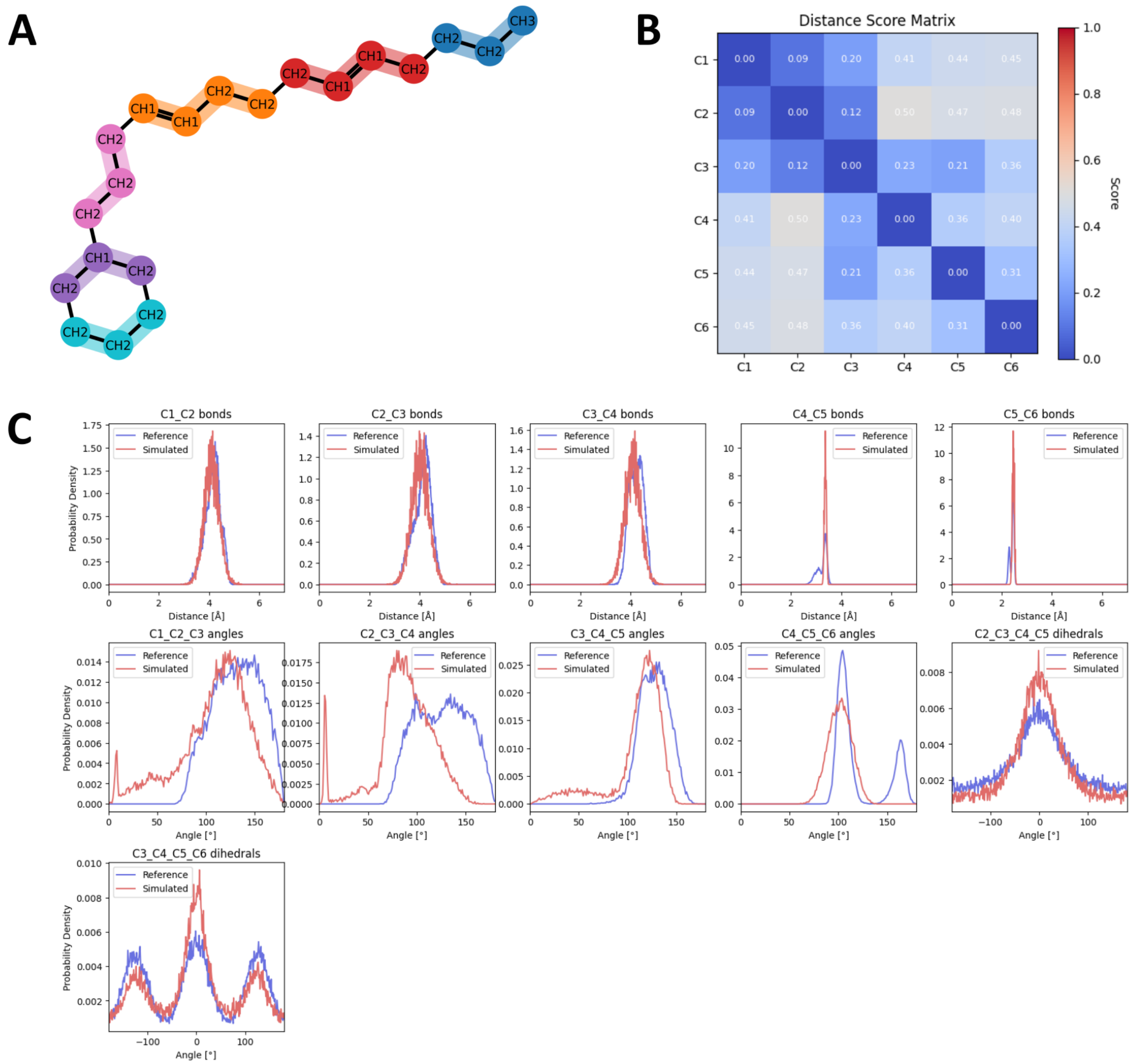

Figure S5: Parametrization of M23. **A:** the mapping of the atomistic structure to CG resolution. **B:** Distance Score Matrix showing distance score for each pairwise distance. **C:** bonded interactions of the pseudo-CG reference trajectory (blue) and distributions generated with CG Simulations (red).

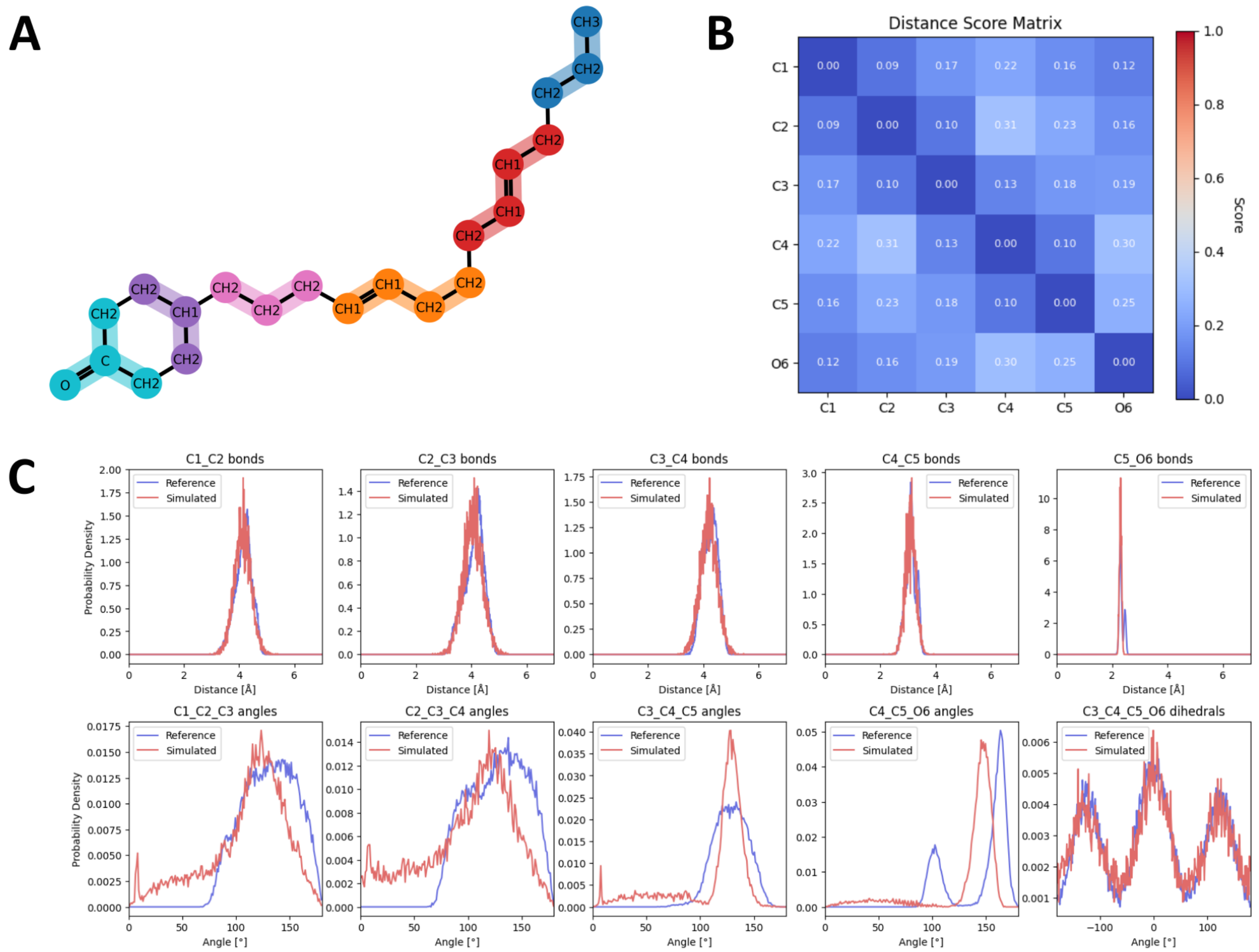

Figure S6: Parametrization of M24. **A:** the mapping of the atomistic structure to CG resolution. **B:** Distance Score Matrix showing distance score for each pairwise distance. **C:** bonded interactions of the pseudo-CG reference trajectory (blue) and distributions generated with CG Simulations (red).

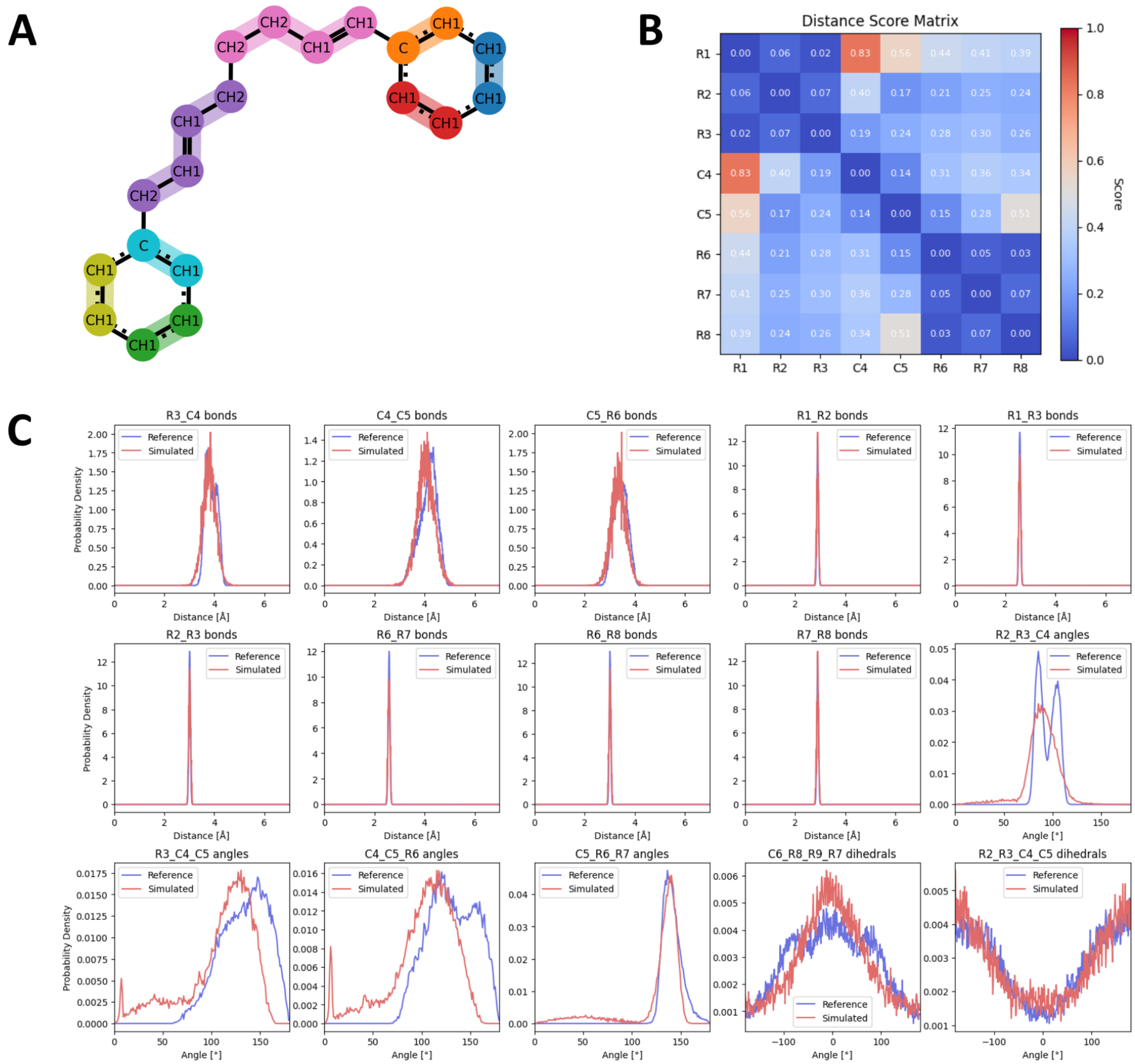

Figure S7: Parametrization of M25. **A:** the mapping of the atomistic structure to CG resolution. **B:** Distance Score Matrix showing distance score for each pairwise distance. **C:** bonded interactions of the pseudo-CG reference trajectory (blue) and distributions generated with CG Simulations (red).

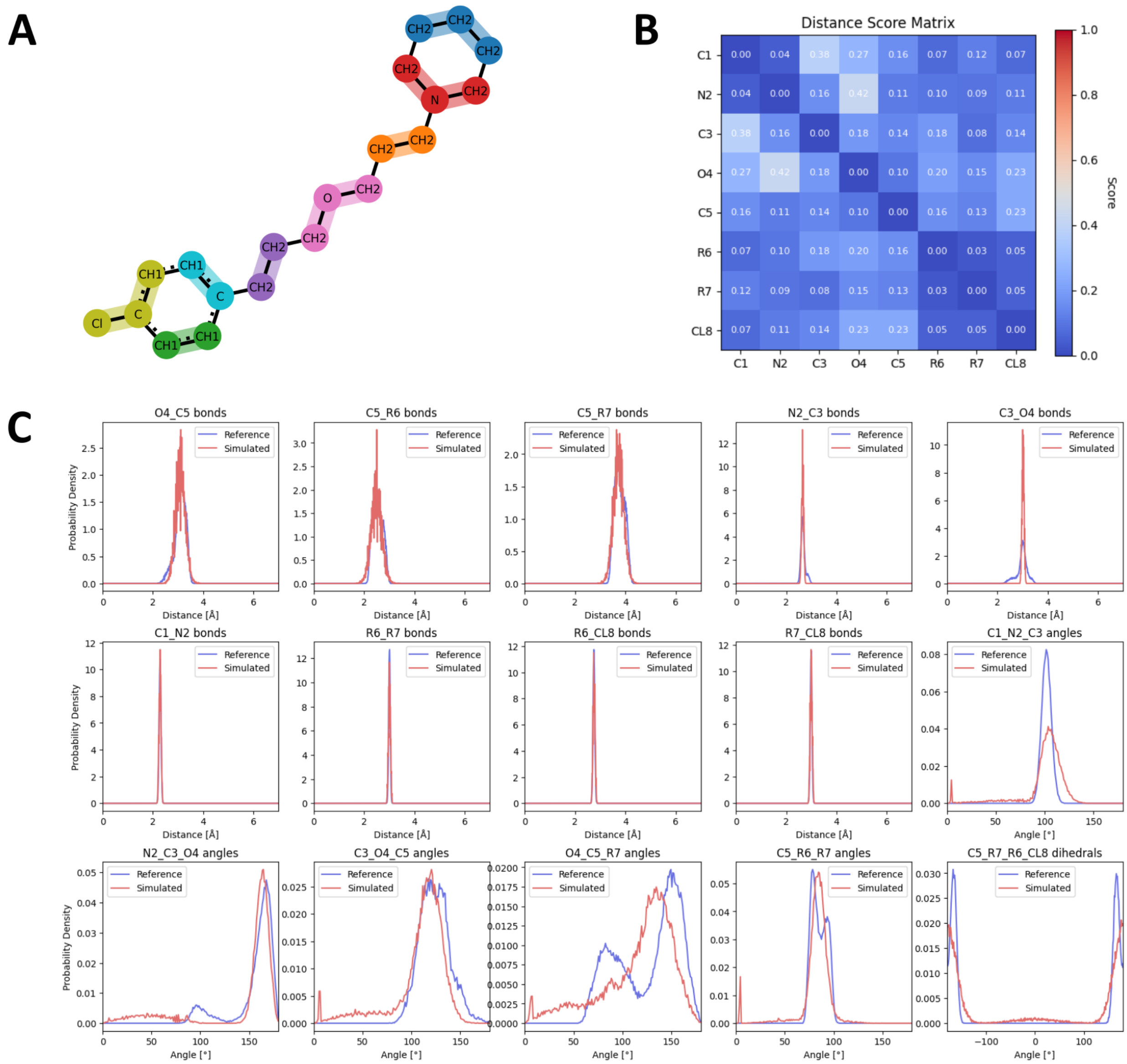

Figure S8: Parametrization of PITO. **A:** the mapping of the atomistic structure to CG resolution. **B:** Distance Score Matrix showing distance score for each pairwise distance. **C:** bonded interactions of the pseudo-CG reference trajectory (blue) and distributions generated with CG Simulations (red).

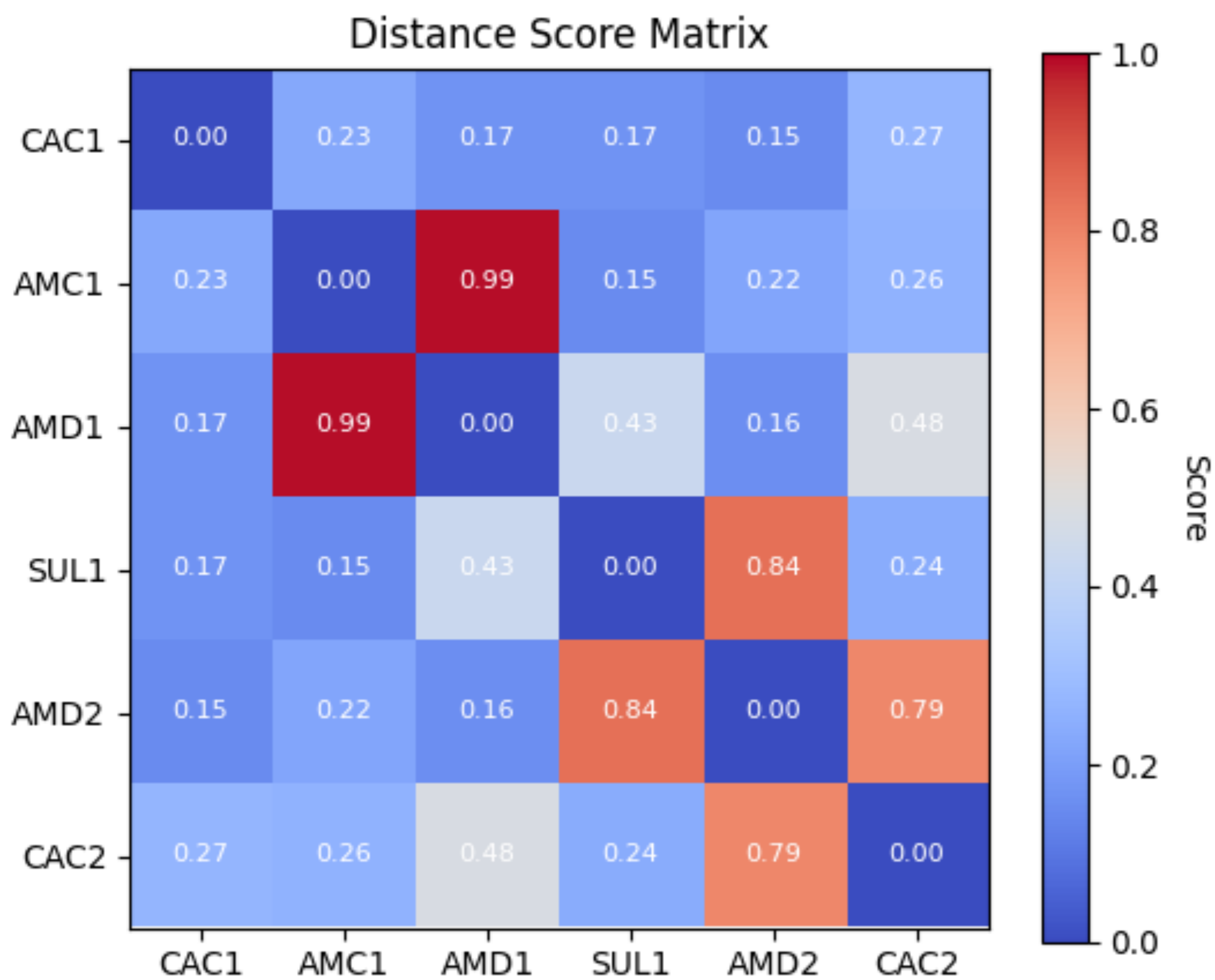

Figure S9: GSH distance score matrix for each pairwise distance. The high values (0.99, 0.94, 0.79) are due to the use of constraints as bonds between adjacent beads in the molecule.

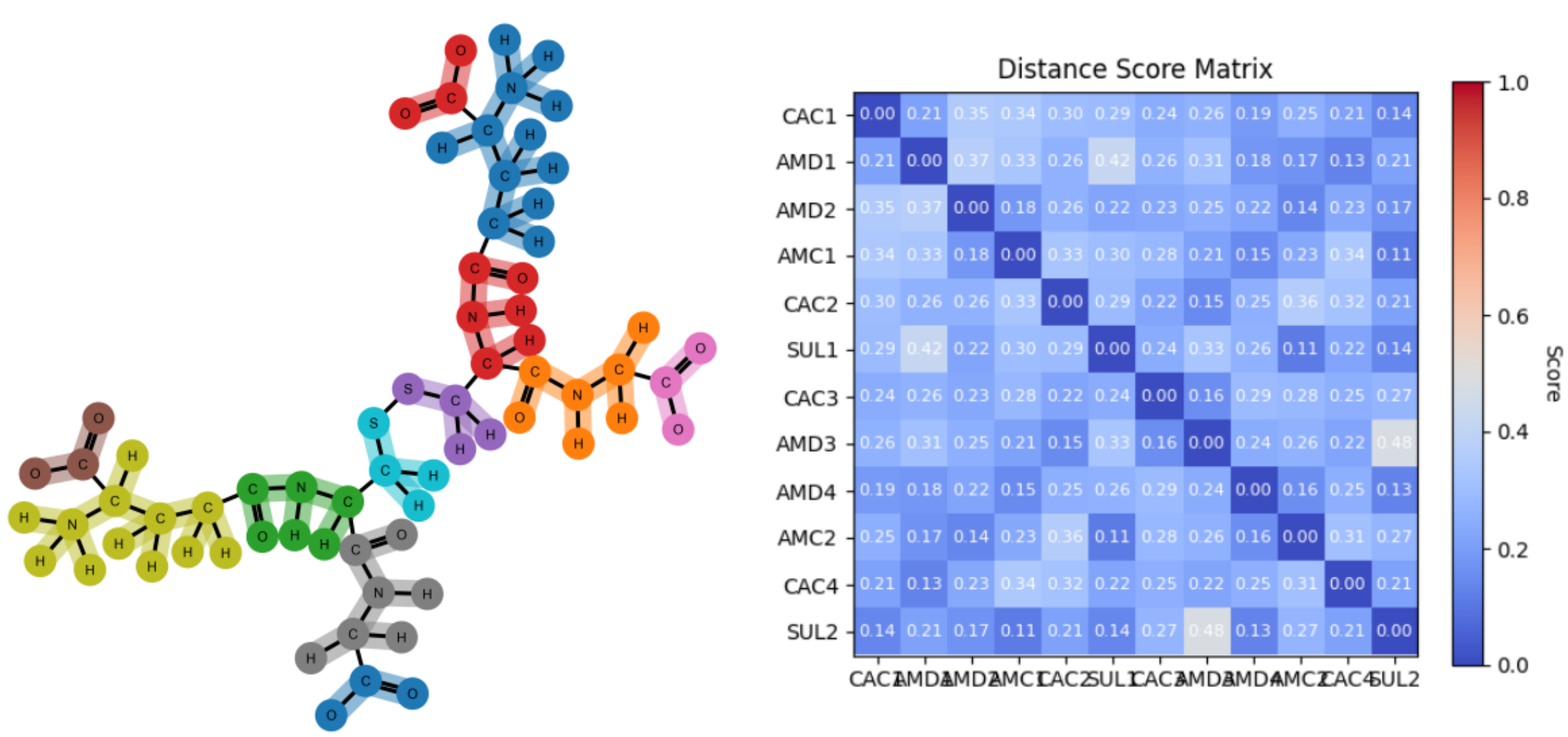

Figure S10. Parameterization of GSSG. Left, mapping of GSSG and the model's distance score matrix for each pairwise distance.

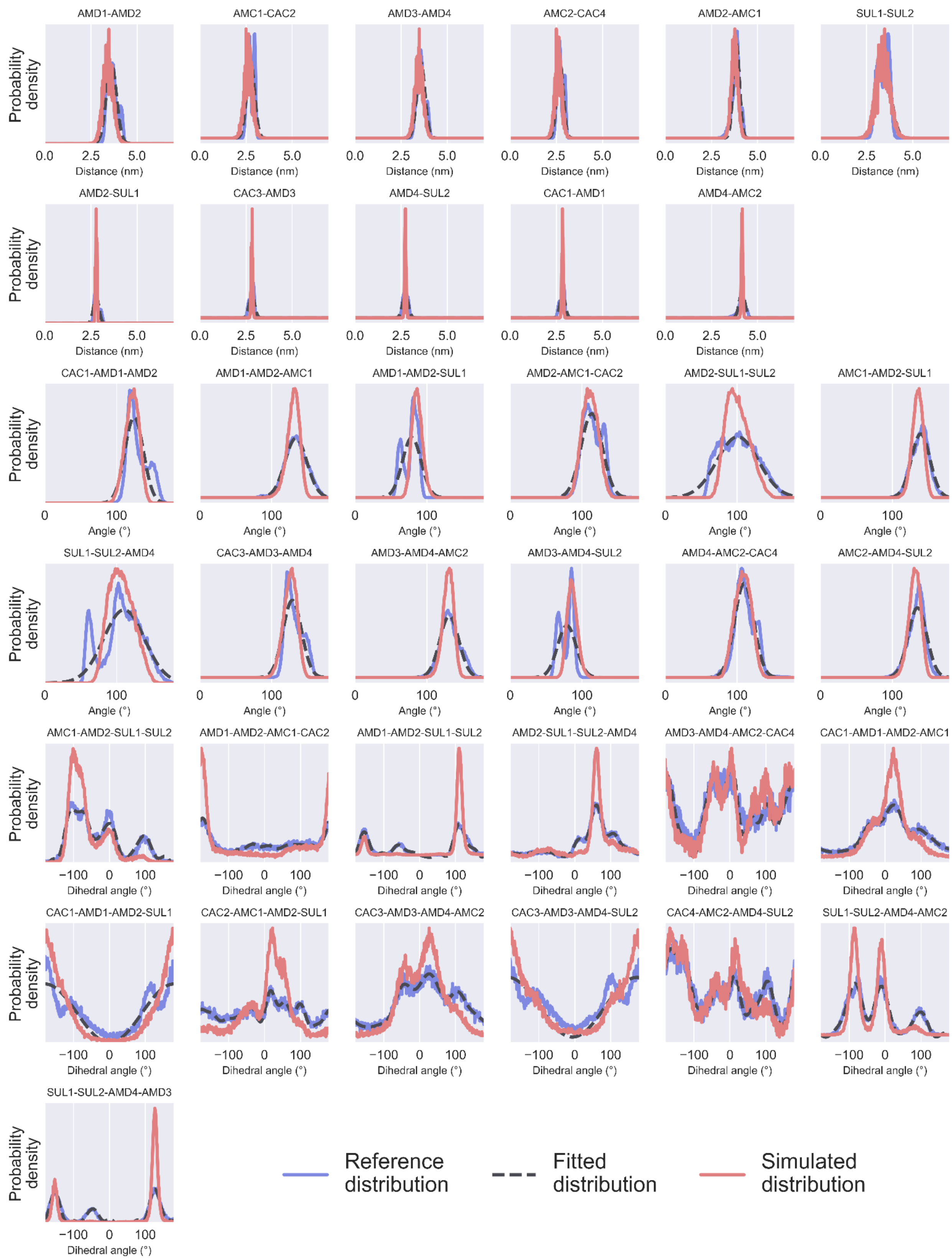

Figure S11: Pseudo-CG, fitted, and CG simulated distributions for GSSG bonded interactions.

4. Polymers

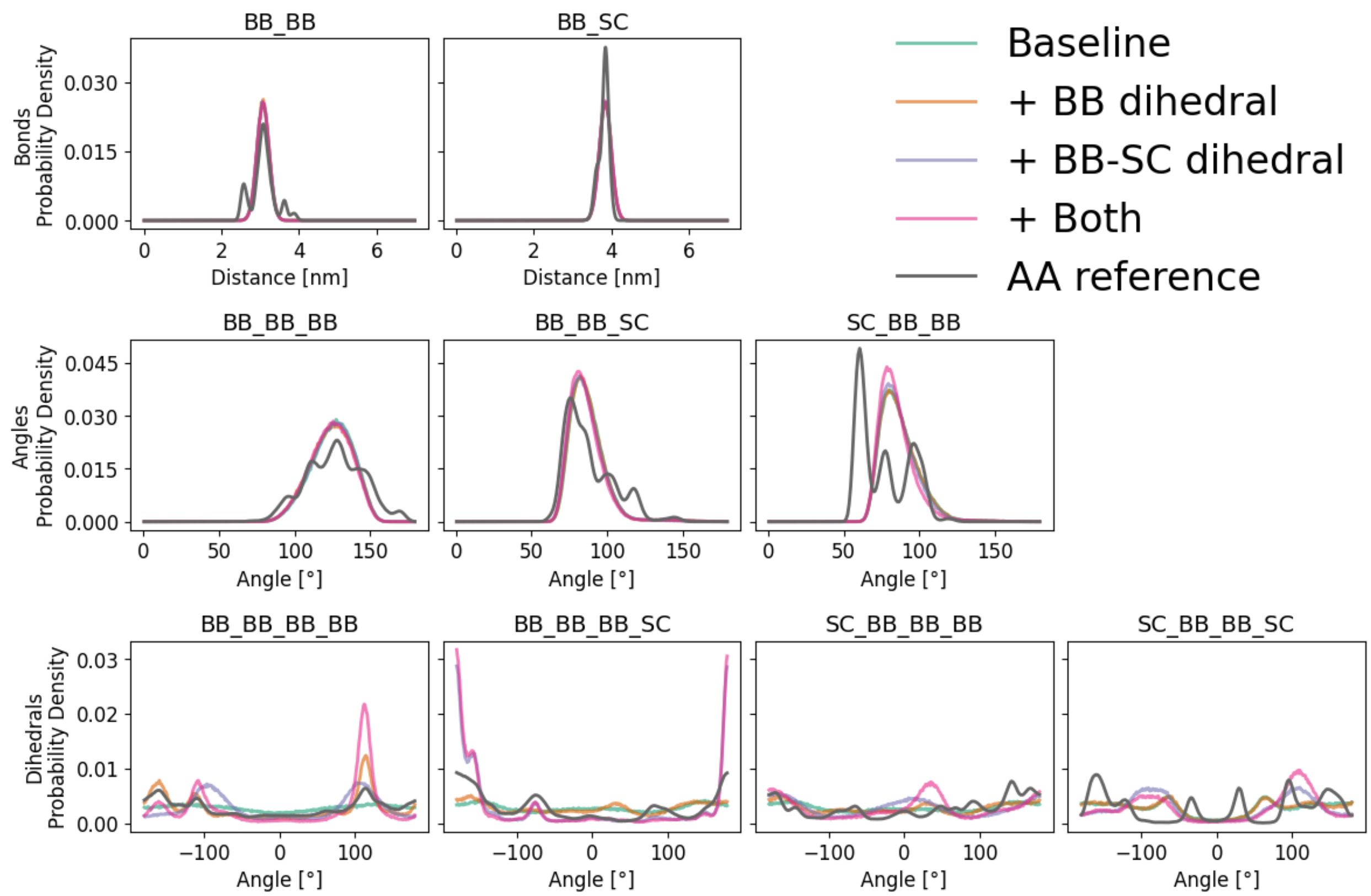

Figure S12: Probability distributions for all bonded interactions the CG models of PMMA in comparison to the atomistic reference distributions. The PMMA mapping consists of a backbone bead (BB) and a sidechain bead (SC). As described in the main text, CG models were generated containing no additional dihedrals (labelled as 'baseline', green line), a dihedral along BB-BB-BB-BB ('+BB dihedral', yellow line), along BB-BB-BB-SC ('+ BB-SC dihedral', purple line), or both ('+ Both', pink line). The pseudo-CG distributions are also plotted ('AA reference, grey line). An additional model containing a SC-BB-BB-SC dihedral was tested but was not developed further due to numerical instability.

Table S1: Scores of bonded interactions across all runs. First Hellinger Distance, second Scoring function. Interactions with a * prefix were only assessed but are not included in the topology and excluded from the averaging in Figure 4.

	**Bonds**	**Angles**	**Dihedrals**
**Baseline:** No dihedral	BB-SC 0.18 (0.17) BB-BB 0.32 (0.22)	BB-BB-BB 0.22 (0.17) SC-BB-BB 0.49 (0.43) BB-BB-SC 0.27 (0.21)	*BB-BB-BB-BB 0.14 (0.1) *BB-BB-BB-SC 0.26 (0.21)
+ BB dihedral	BB-SC 0.18 (0.17) BB-BB 0.32 (0.22)	BB-BB-BB 0.22 (0.17) SC-BB-BB 0.49 (0.42) BB-BB-SC 0.26 (0.21)	BB-BB-BB-BB 0.11 (0.08) *BB-BB-BB-SC 0.26 (0.21)
+ BB-SC dihedral	BB-SC 0.18 (0.17) BB-BB 0.32 (0.22)	BB-BB-BB 0.22 (0.17) SC-BB-BB 0.49 (0.42) BB-BB-SC 0.26 (0.21)	*BB-BB-BB-BB 0.24 (0.18) BB-BB-BB-SC 0.26 (0.2)
+ Both dihedrals	BB-SC 0.19 (0.15) BB-BB 0.32 (0.22)	BB-BB-BB 0.22 (0.18) SC-BB-BB 0.51 (0.42) BB-BB-SC 0.26 (0.22)	BB-BB-BB-BB 0.28 (0.23) BB-BB-BB-SC 0.31 (0.25)

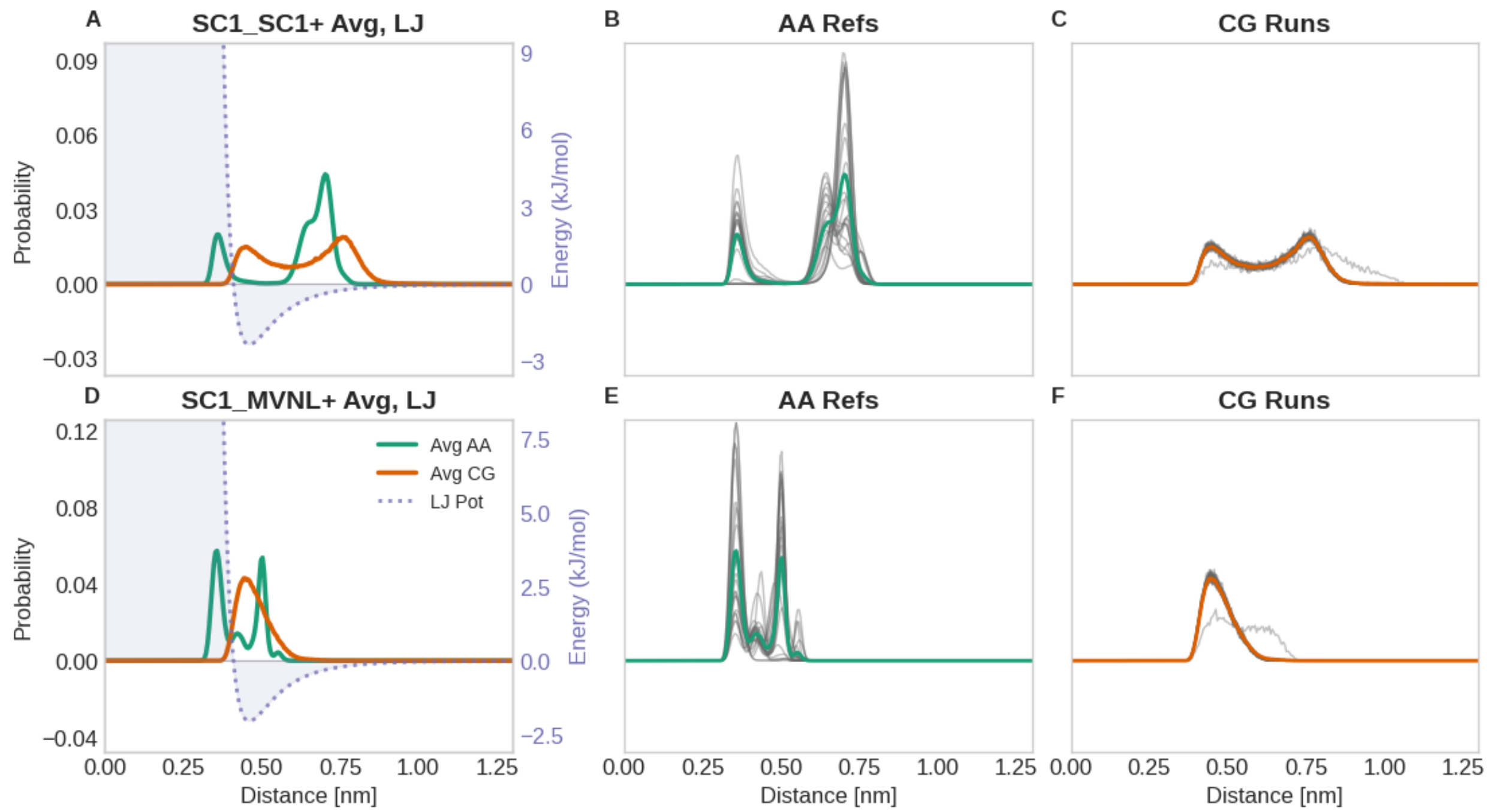

Figure S13: bead-interaction specific LJ potentials hinder full match of the atomistic reference.1-3 and 1-4 Distances are geometrically hindered by large bead radiuses. The full range of 1-3 distances (A-C), and 1-4 distances (D-F) is not captured by the coarse-grained polymer model. The gray lines are the individual distances across the polymer, while averages are depicted in colour. The reasons are the Lennard-Jones (LJ) potentials of the interacting bead types from the Martini3 force-field. They provide a large energy barrier for the distributions to match completely. However, for sake of compatibility with other subsequent models the LJ potentials were not changed to provide an improved description of the polymer model.

Reference Generation

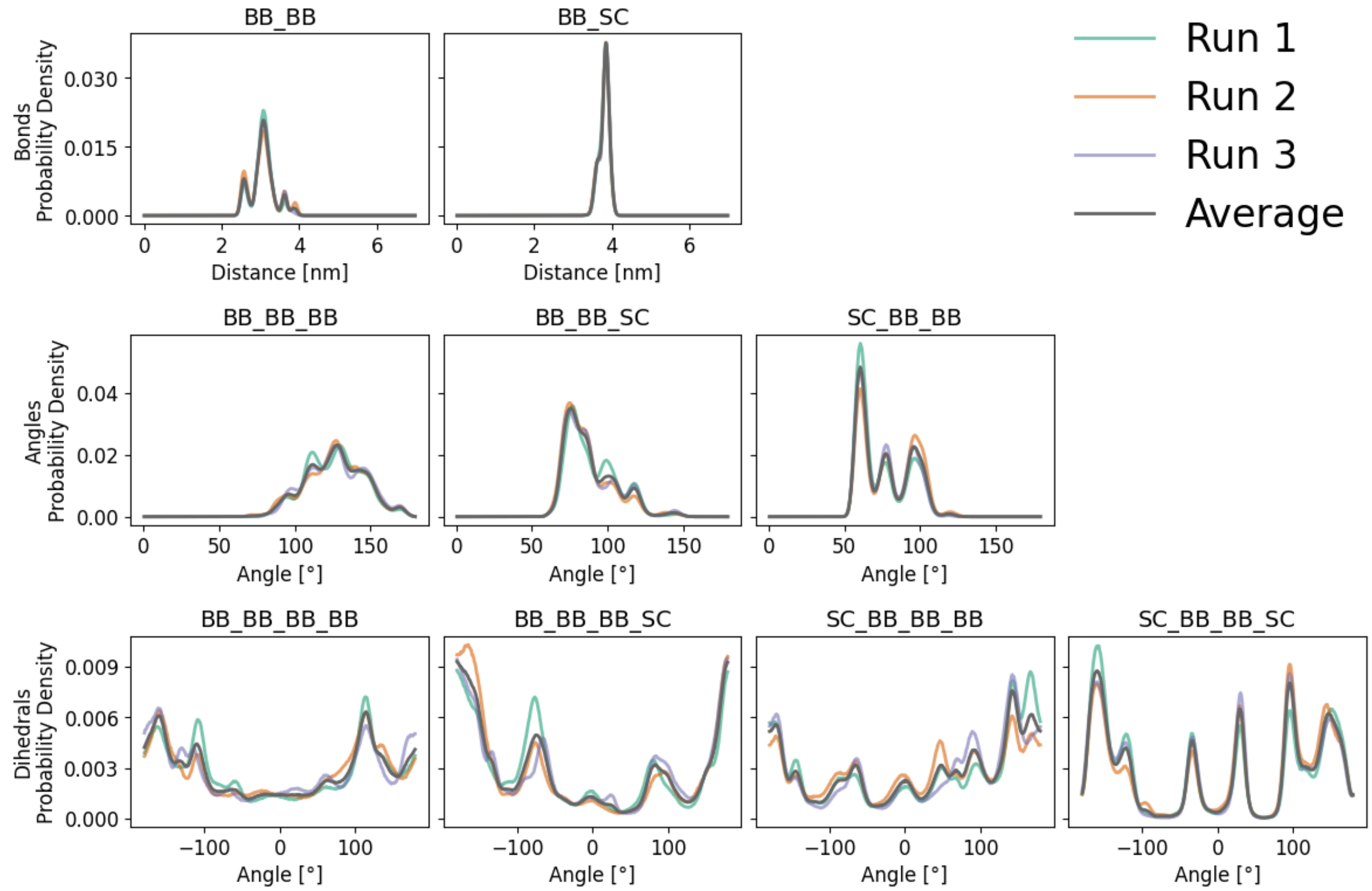

Figure S14: combination of 3 individual atomistic reference simulations into one average atomistic reference across all relevant bonded interactions.

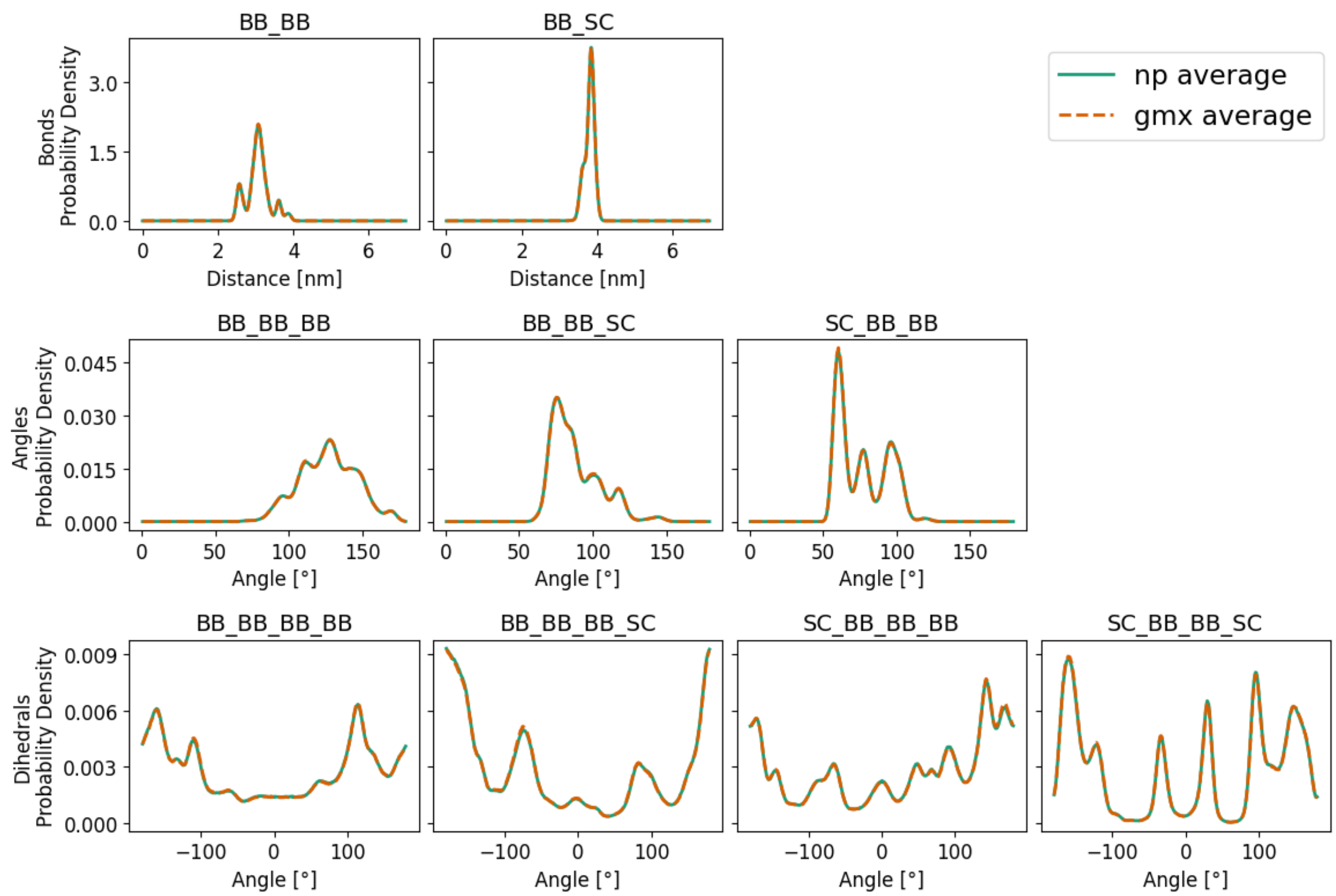

Figure S15: assessing potential pitfalls of averaging techniques. The average of each distribution shown in Figure S14 was taken using two different methods. First, the arithmetic mean was computed from the distributions as taken from each individual trajectory. Second, $\frac{2}{3}$ of a trajectory was sampled at random and combined. The average was then taken over the concatenated trajectory. The 'np average' depicts the average over each individual property across the three reference simulations. The 'gmx average' represents the approach via concatenating a part of each simulated trajectory and calculating the individual interaction distributions from this combined trajectory.

5. Mapping format style

.map style

An example of the .map style is shown below. The file contains three directives, [molecule], [martini], and [atoms]. In the example below, a semicolon indicates a comment that will not be parsed.

The [molecule] directive contains the name of the residue at atomistic and CG resolution. The map file here is designed for a residue named "LIG" at atomistic resolution, which will be mapped to a residue called "GSH" at CG resolution.

The [martini] directive simply contains a list of the names of the beads which this residue is to be mapped to.

The [atoms] directive contains an entry for each of the atoms in the residue. Each atom has an index for the entry, the name of the atom, and the name of the CG bead which it is to be mapped to.

```
[ molecule ]
LIG GSH ; resname_from resname_to

[ martini ]
CAC1 AMC1 AMD1 SUL1 AMD2 CAC2 ; names of CG beads

[ atoms ]
1     N1    AMC1 ; atomindex, atomistic atomname, CG bead name
2     C1    AMC1
3     C2    AMC1
4     C3    AMC1
5     C4    AMD1
6     O1    AMD1
7     N2    AMD1
8     C5    AMD1
9     C6    SUL1
10    S1    SUL1
11    C7    AMD2
12    O2    AMD2
13    N3    AMD2
```

```
14   C8   AMD2
15   C9   CAC2
16   O3   CAC2
17   O4   CAC2
18   C10  CAC1
19   O5   CAC1
20   O6   CAC1
21   H1   AMC1
22   H2   AMC1
23   H3   AMC1
24   H4   AMC1
25   H5   AMC1
26   H6   AMC1
27   H7   AMC1
28   H8   AMC1
29   H9   AMD1
30   H10  AMD1
31   H11  SUL1
32   H12  SUL1
33   H13  SUL1
34   H14  AMD2
35   H15  AMD2
36   H16  AMD2
```

CGsmiles style

Fast-Forward accepts mappings described by CGsmiles strings. The example below shows an example for a Martini lipid with a sugar headgroup. CGsmiles strings may be provided with three or two resolutions. Three resolutions describe the residues, Martini beads, and all-atom molecules while two resolutions are assumed to just describe the Martini to all-atom mapping. In the second case the molecule is considered a single residue with a generic resname.

Mapping definitions via CGsmiles allow for some special features not accessible via the other formats. In particular, using the annotation syntax weights can be given to each atom chaning by how much the atom contributes to the specific position. Furthermore, atoms can be split between residues as the CGsmiles string directly matches the all-atom graph.

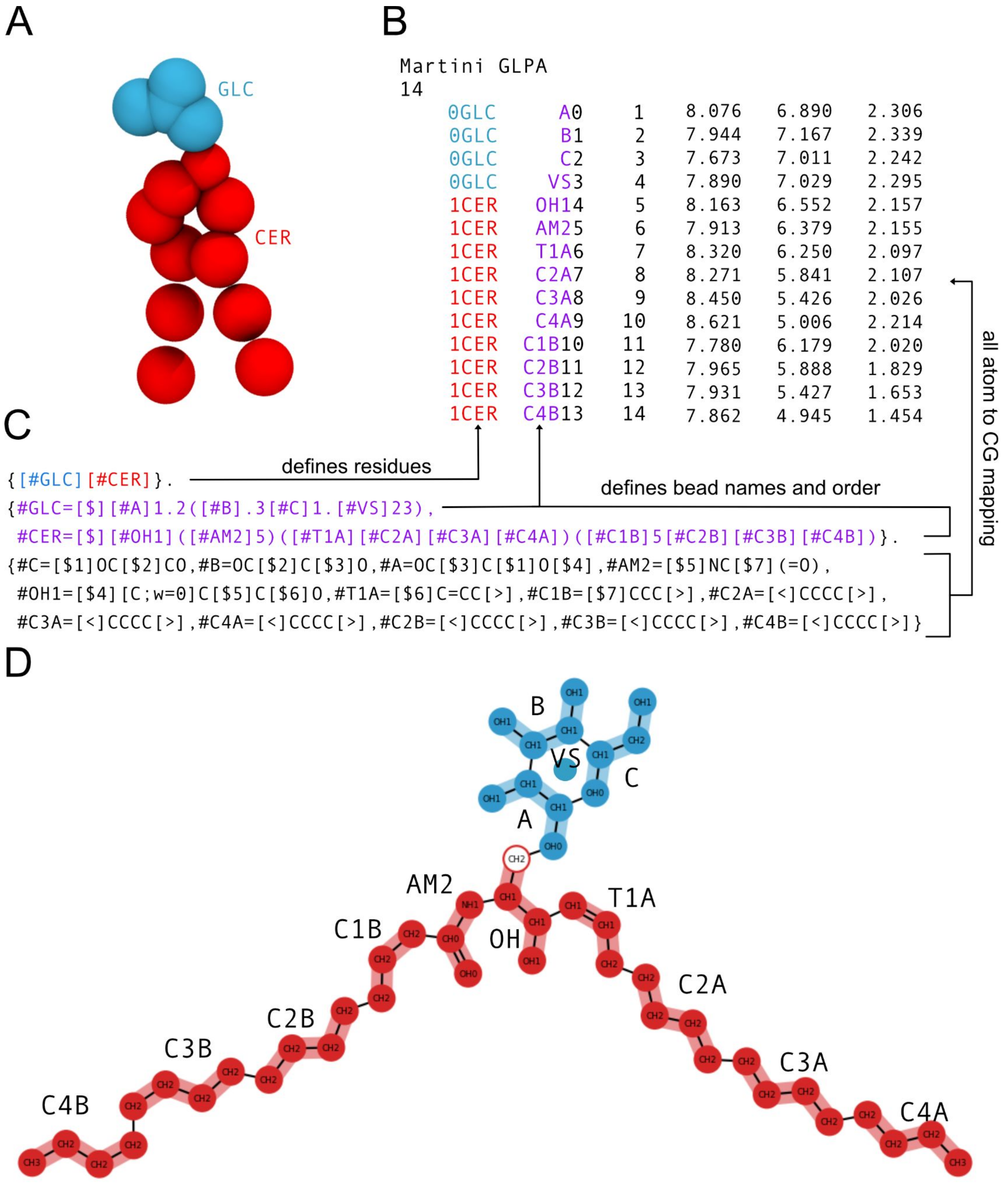

Figure S16: CGsmiles string for mapping of GLPA. The 3D structure of GLPA (A) as defined in a GRO file (B), can be mapped from the all-atom structure using a CGsmiles string (C). The CGsmiles string has 3 levels enclosed in curly braces. The first level describes the number of residues in the molecule and their name (GLC & CER respectively). The next level described the Martini beads that these residues contain as well as how the Martini beads connect. In this case the 'A' bead in GLC connects to the \`OH\` in CER. The bead names and the order defines their

definition in the GRO file. Note that each beads is always appended by an index running from 0-N. This ensures the bead names are always unique, which may be needed for other programs in the Martini Ecoystem. Finally, the third level defines the all-atom to Martini mapping for each bead and how these fragments connect according to the previously defined mapping (D). Each atom can be given a weight using the 'w' key within the CGsmiles annotation syntax (e.g. OH bead in GLPA).

Table S2: Mapping specifications and features

	rename residues	map across residue boundaries	weights	format documentation
.map	yes	no	yes	[1]
.mapping	yes	yes but not supported	yes	[1]
CGsmiles string	yes	yes	yes	[2]

[1] https://vermouth-martinize.readthedocs.io/en/latest/file_formats.html#mapping-files-map-mapping
[2] https://cgsmiles.readthedocs.io/en/latest/syntax/introduction.html
